# Supplementary material for: MOSTWAS: Multi-Omic Strategies for Transcriptome-Wide Association Studies
Source: PLoS Genet. 2021 Mar 8;17(3):e1009398. doi: 10.1371/journal.pgen.1009398 (PMC7971899; doi:10.1371/journal.pgen.1009398)
Supplement: S1 Fig — (A) Proportion of gene-trait associations at P<2.5×10^−6^ using local-only (red), BGW-TWAS (blue), and the most predictive MOSTWAS (green) models across various local and distal expression heritability, trait heritability, and two setting of causal eQTL proportion. (B) Proportion of significant gene-trait associations across the same simulation parameters with no distal effect on the trait in the simulated external GWAS panel. (PDF) [file pgen.1009398.s002.pdf]

MOSTWAS: Multi-omic strategies for transcriptome-wide association studies

Supplemental Figures

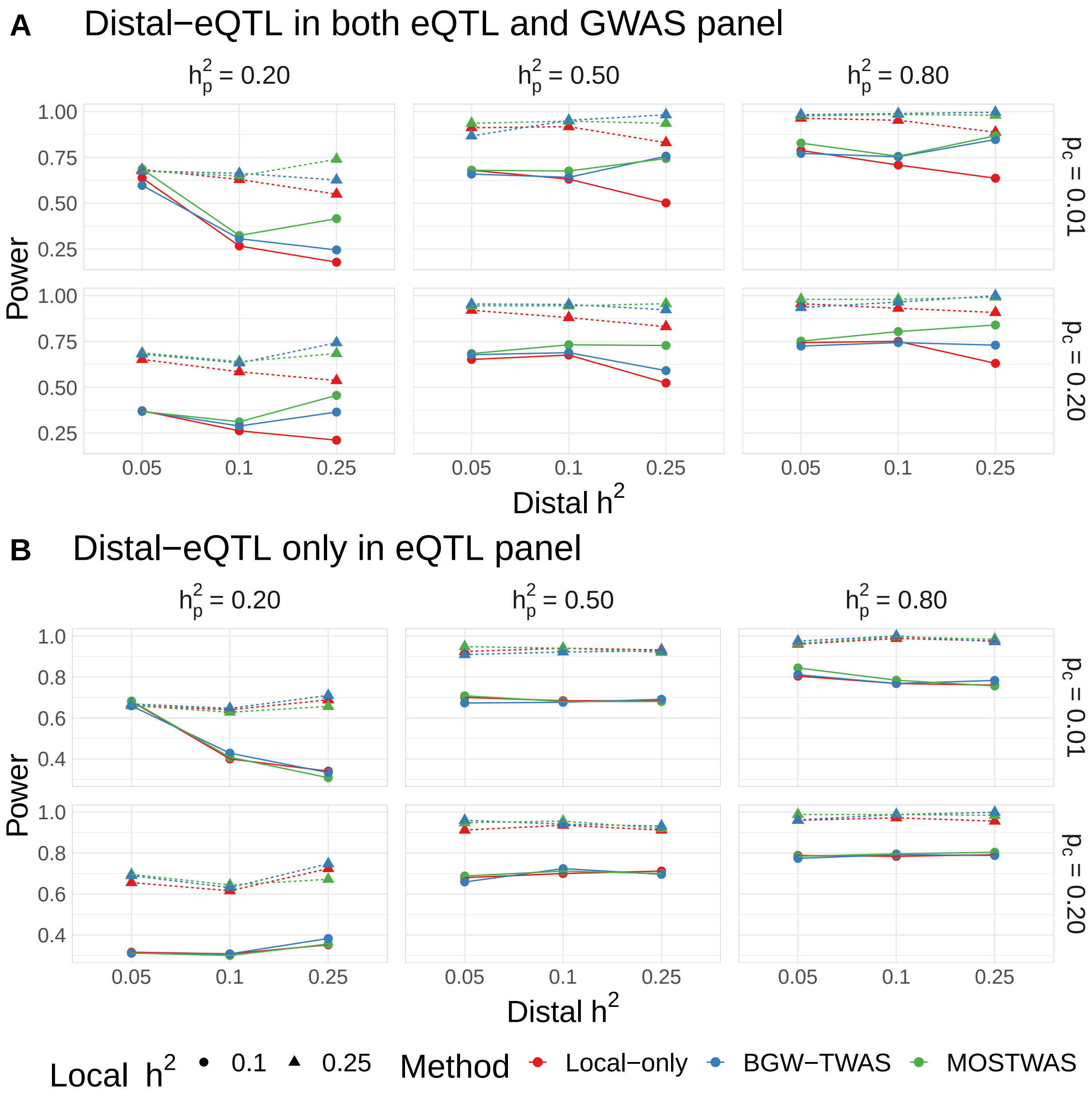

Figure S1: *Comparison of TWAS power via simulations using MOSTWAS, BGW-TWAS, and local-only models.* (A) Proportion of gene-trait associations at $P < 2.5 \times 10^{-6}$ using local-only (red), BGW-TWAS (blue), and the most predictive MOSTWAS (green) models across various local and distal expression heritabilities, trait heritability, and two setting of causal eQTL proportion. (B) Proportion of significant gene-trait associations across the same simulation parameters with no distal effect on the trait in the simulated external GWAS panel.
