# Supplementary material for: MOSTWAS: Multi-Omic Strategies for Transcriptome-Wide Association Studies
Source: PLoS Genet. 2021 Mar 8;17(3):e1009398. doi: 10.1371/journal.pgen.1009398 (PMC7971899; doi:10.1371/journal.pgen.1009398)
Supplement: S2 Fig — Mean adjusted R2 across various local and distal expression heritability, trait heritability, and causal proportions using local-only (red) and the best MOSTWAS (blue) models. The error bars reflect a width of 1 standard deviation of the 1,000 simulated adjusted R2 values. (PDF) [file pgen.1009398.s003.pdf]

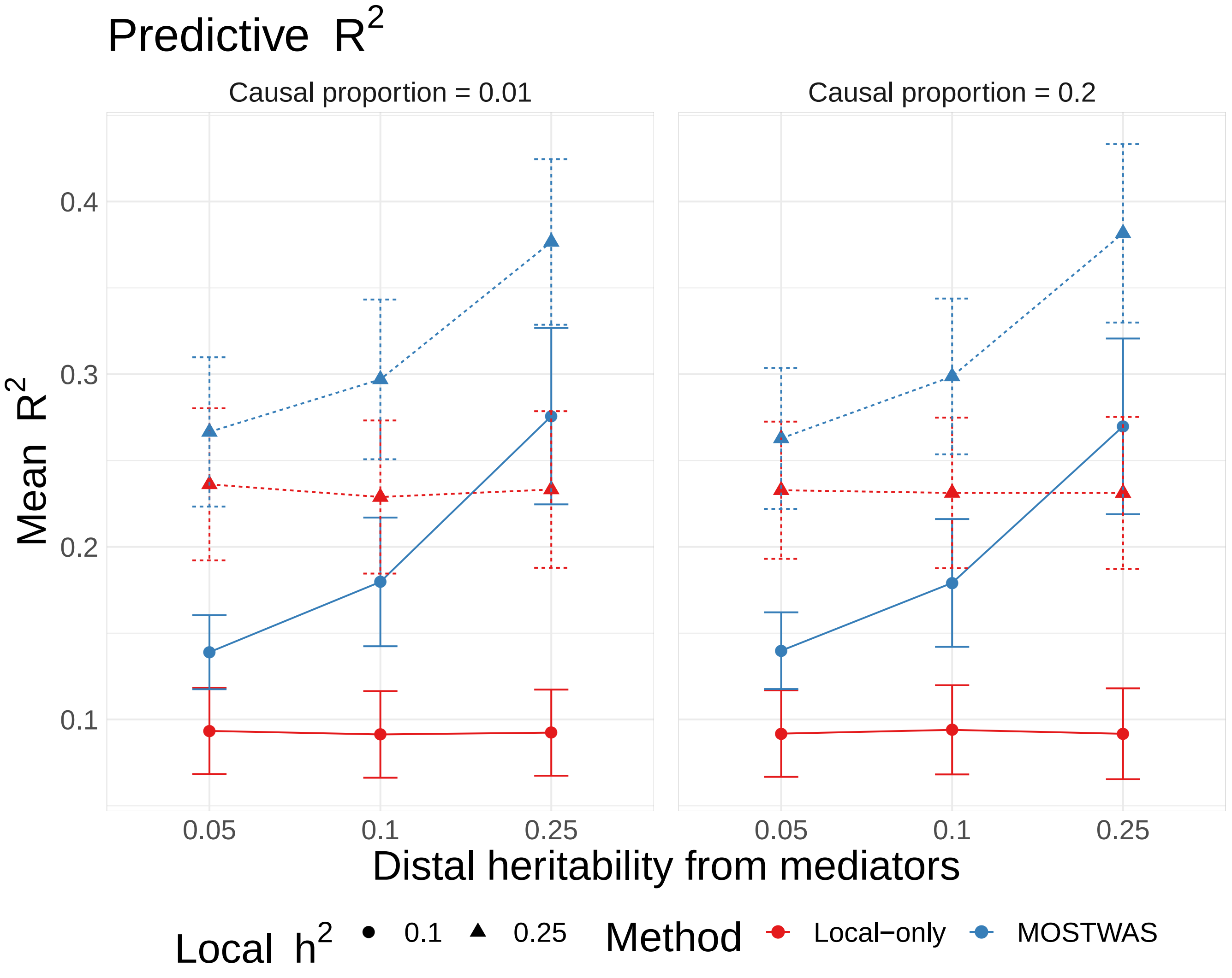

Figure S2: *Comparison of predictive* R^2 *in simulations.* Mean adjusted R^2 across various local and distal expression heritabilities, trait heritabilities, and causal proportions using local-only (red) and the best MOSTWAS (blue) models. The error bars reflect a width of 1 standard deviation of the 1,000 simulated adjusted R^2 values.
