# Supplementary material for: MOSTWAS: Multi-Omic Strategies for Transcriptome-Wide Association Studies
Source: PLoS Genet. 2021 Mar 8;17(3):e1009398. doi: 10.1371/journal.pgen.1009398 (PMC7971899; doi:10.1371/journal.pgen.1009398)
Supplement: S3 Fig — Boxplots of false positive rate (Y -axis) across various distal expression heritability settings (X-axis), stratified by local expression heritability (horizontal) and causal eQTL proportion (vertically) and colored by the method used to test gene-trait associations. These boxplots are from 20 simulations of 1,000 permuted phenotype traits in the simulated GWAS. The red line provides a reference at a false positive rate of 0.05. (PDF) [file pgen.1009398.s004.pdf]

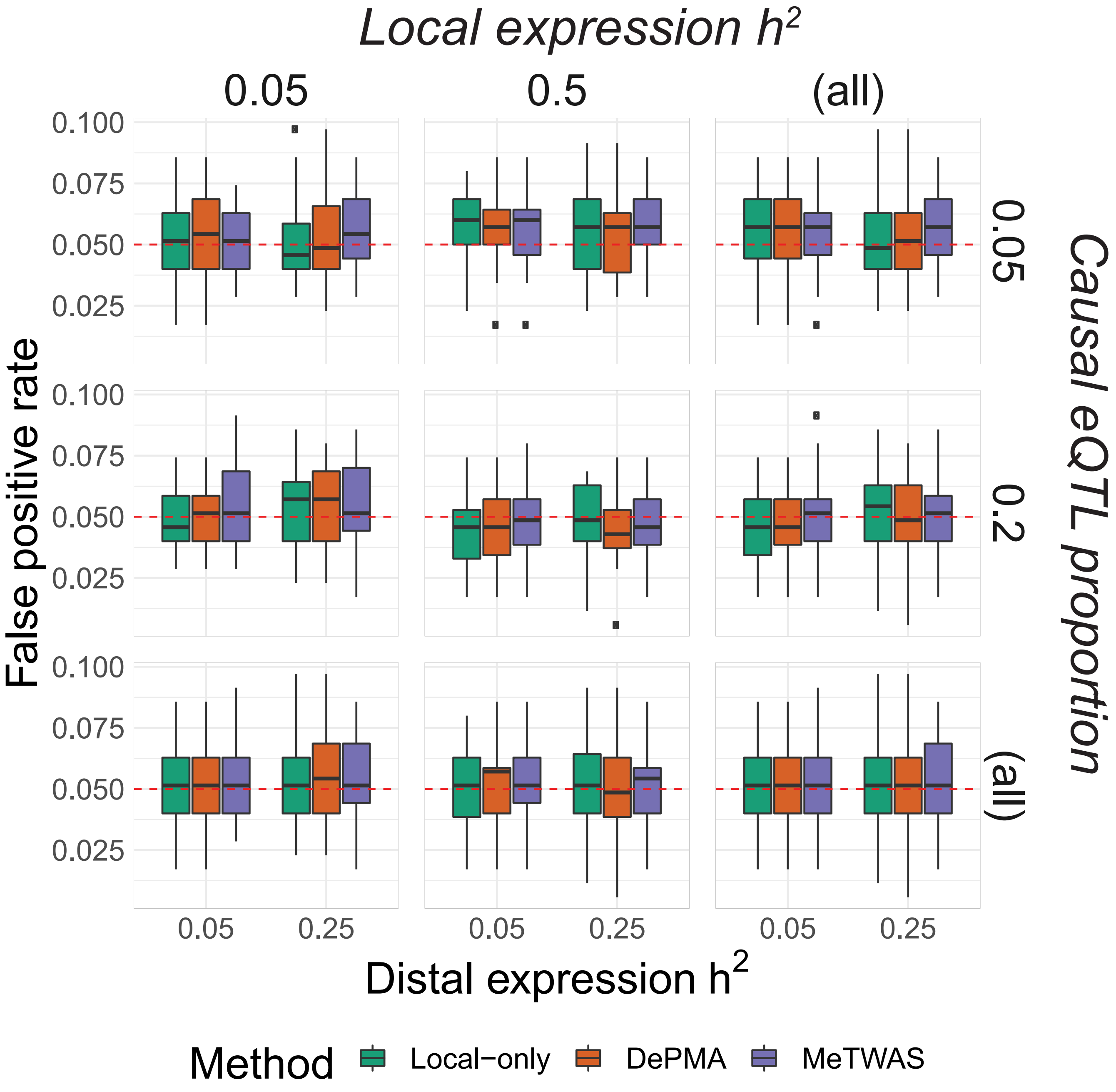

Figure S3: *Comparison of false positive rates in simulations.* Boxplots of false positive rate (Y-axis) across various distal expression heritability settings (X-axis), stratified by local expression heritability (horizontal) and causal eQTL proportion (vertically) and colored by the method used to test gene-trait associations. These boxplots are from 20 simulations of 1,000 permuted phenotype traits in the simulated GWAS. The red line provides a reference at a false positive rate of 0.05.
