# Supplementary material for: MOSTWAS: Multi-Omic Strategies for Transcriptome-Wide Association Studies
Source: PLoS Genet. 2021 Mar 8;17(3):e1009398. doi: 10.1371/journal.pgen.1009398 (PMC7971899; doi:10.1371/journal.pgen.1009398)
Supplement: S4 Fig — Across various sample sizes for the eQTL reference (X-axis) panel and GWAS imputation panel (color), the power of the distal added-last test to detect a significant association with distal variants conditional on a significant local association at FDR-adjusted P<0.05. (PDF) [file pgen.1009398.s005.pdf]

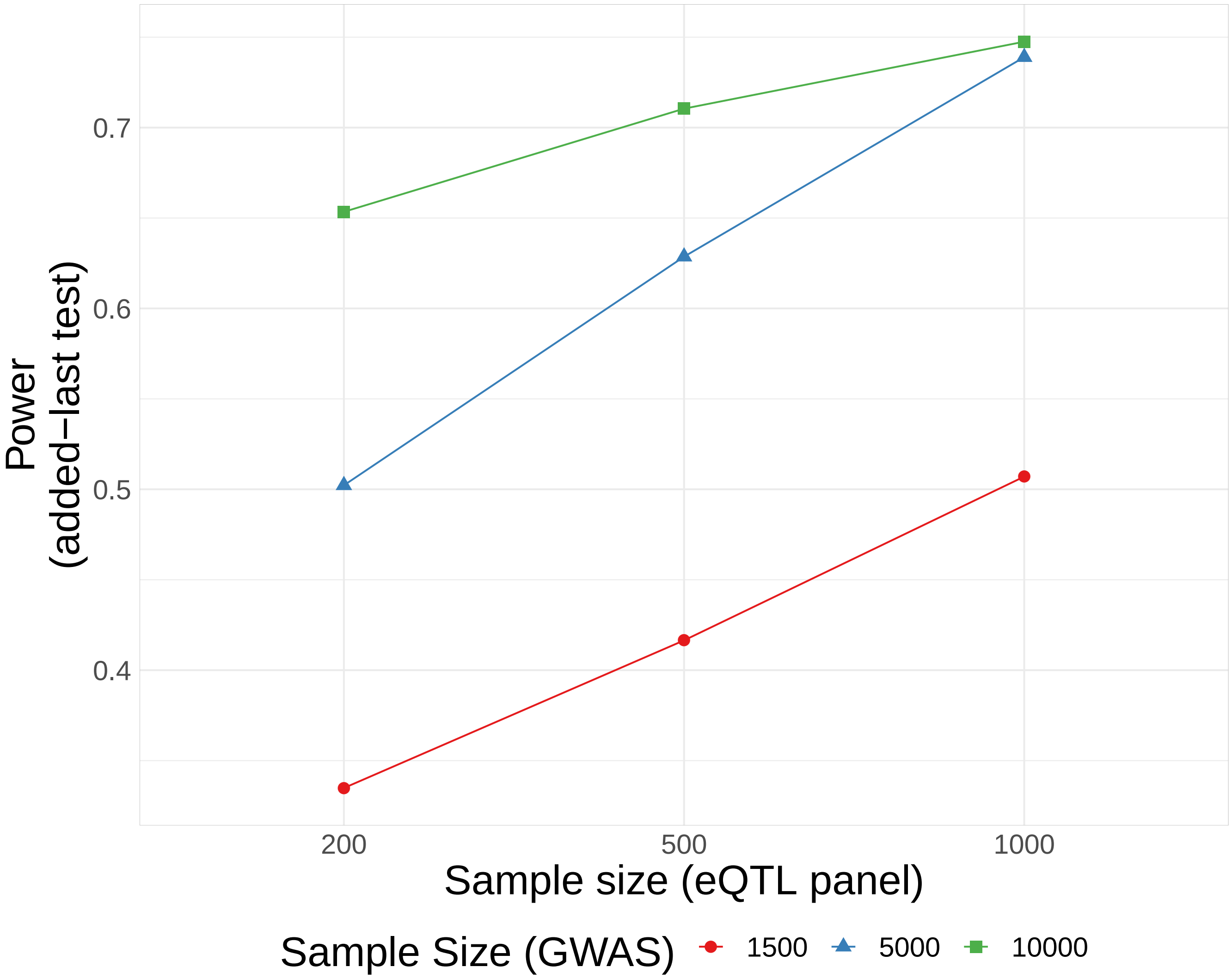

Figure S4: *Simulation analysis for the power of the distal variants added-last test.* Across various sample sizes for the eQTL reference (X-axis) panel and GWAS imputation panel (color), the power of the distal added-last test to detect a significant association with distal variants conditional on a significant local association at FDR-adjusted $P < 0.05$.
