# Supplementary material for: MOSTWAS: Multi-Omic Strategies for Transcriptome-Wide Association Studies
Source: PLoS Genet. 2021 Mar 8;17(3):e1009398. doi: 10.1371/journal.pgen.1009398 (PMC7971899; doi:10.1371/journal.pgen.1009398)
Supplement: S5 Fig — Z-statistic of TWAS association on the Y-axis and chromosomal position of gene on X-axis. Genes are colored red if overall P<2.5×10^−6^ and nominal permutation P<0.05 and labelled if distal association is significant at a Bonferroni threshold (α=0.0518=0.0028). (PDF) [file pgen.1009398.s006.pdf]

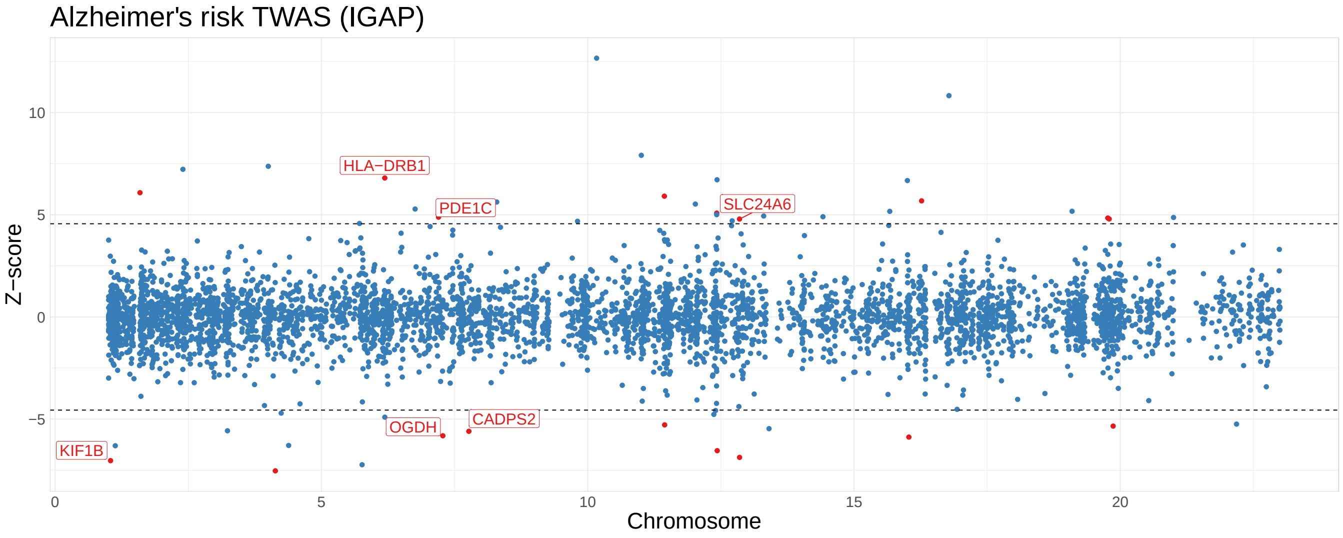

Figure S5: *Manhattan plot for Alzheimer's risk associations using MOSTWAS on IGAP summary statistics.* Z-statistic of TWAS association on the Y-axis and chromosomal position of gene on X-axis. Genes are colored red if overall $P < 2.5 \times 10^{-6}$ and nominal permutation $P < 0.05$ and labelled if distal association is significant at a Bonferroni threshold ($\alpha = 0.05/18 = 0.0028$).
