# Supplementary material for: MOSTWAS: Multi-Omic Strategies for Transcriptome-Wide Association Studies
Source: PLoS Genet. 2021 Mar 8;17(3):e1009398. doi: 10.1371/journal.pgen.1009398 (PMC7971899; doi:10.1371/journal.pgen.1009398)
Supplement: S6 Fig — −log10 P-values of weighted burden gene-trait associations using PGC MDD risk GWAS in predominantly European-ancestry patients (A) and iCOGs survival GWAS in European-ancestry women (B) and among genes that were predicted at cross-validation R2≥0.01 using both local-only and MOSTWAS models and have enough SNPs in summary statistics to conduct TWAS via weighted burden test. The X- and Y-axes display the −log10 P-values for local-only and the best MOSTWAS model, respectively. Points are colored black if P-value of association is less than or equal using the MOSTWAS model. The horizontal and vertical reference lines indicate overall Bonferroni-corrected significance thresholds (P<2.5×10^−6^). (PDF) [file pgen.1009398.s007.pdf]

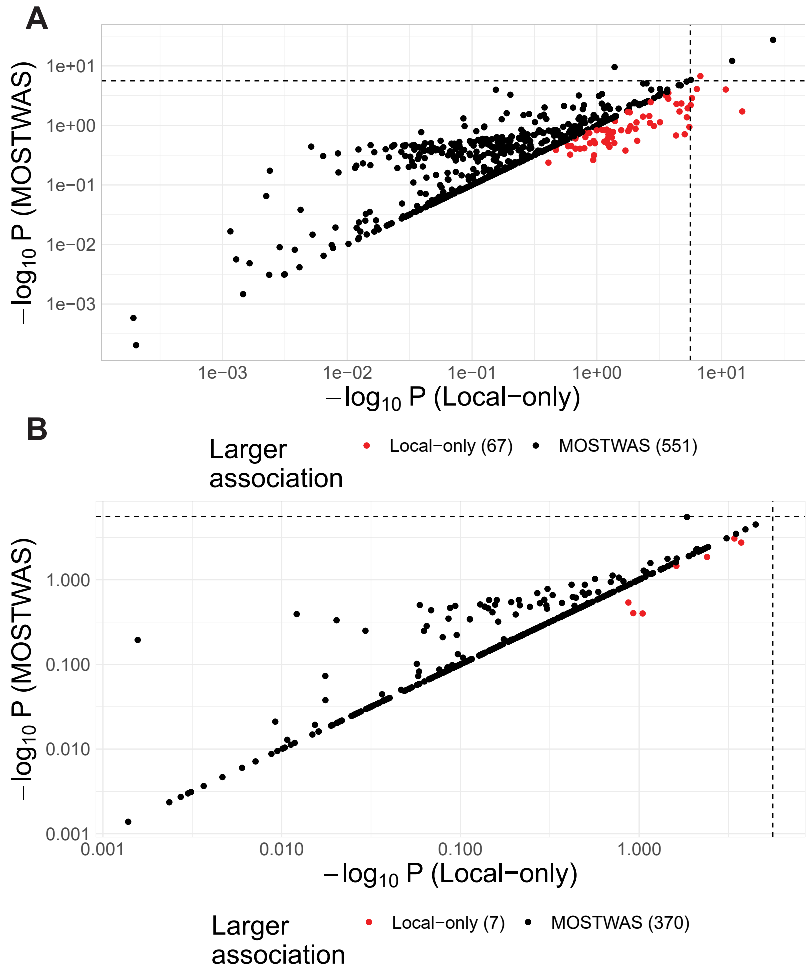

Figure S6: *Gene-trait associations in iCOGs and PGC using local-only and MOSTWAS models.* $-log_{10}P$-values of weighted burden gene-trait associations using PGC MDD risk GWAS in predominantly European-ancestry patients (A) and iCOGs survival GWAS in European-ancestry women (B) and among genes that were predicted at cross-validation $R^2 \geq 0.01$ using both local-only and MOSTWAS models and have enough SNPs in summary statistics to conduct TWAS via weighted burden test. The X- and Y-axes display the $-log_{10}P$-values for local-only and the best MOSTWAS model, respectively. Points are colored black if P-value of association is less than or equal using the MOSTWAS model. The horizontal and vertical reference lines indicate overall Bonferroni-corrected significance thresholds ($P < 2.5 \times 10^{-6}$)
