# Supplementary material for: MOSTWAS: Multi-Omic Strategies for Transcriptome-Wide Association Studies
Source: PLoS Genet. 2021 Mar 8;17(3):e1009398. doi: 10.1371/journal.pgen.1009398 (PMC7971899; doi:10.1371/journal.pgen.1009398)
Supplement: S7 Fig — QQ-plots of Z scores from TWAS for MDD in PGC (A) and breast cancer-specific survival in iCOGs (B) with local-only models (left) and MOSTWAS (right) (PDF) [file pgen.1009398.s008.pdf]

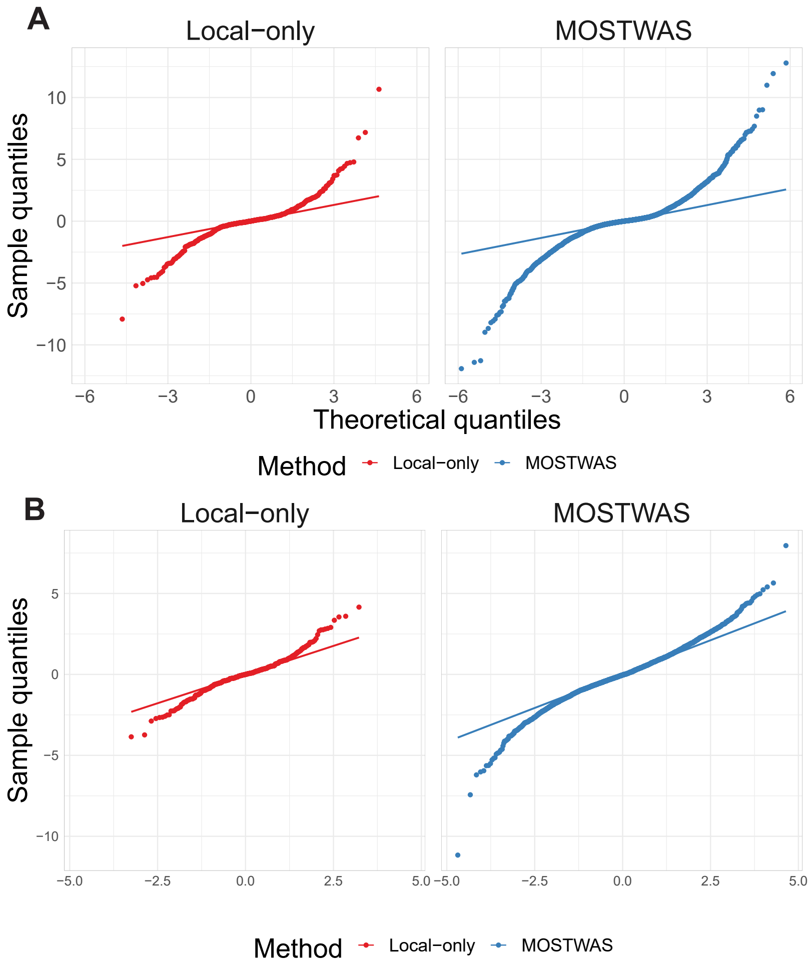

Figure S7: *Comparison of QQ-plots from TWAS associations.* QQ-plots of Z scores from TWAS for MDD in PGC (A) and breast cancer-specific survival in iCOGs (B) with local-only models (left) and MOSTWAS (right)
