# Supplementary material for: MOSTWAS: Multi-Omic Strategies for Transcriptome-Wide Association Studies
Source: PLoS Genet. 2021 Mar 8;17(3):e1009398. doi: 10.1371/journal.pgen.1009398 (PMC7971899; doi:10.1371/journal.pgen.1009398)
Supplement: S8 Fig — QQ-plots of −log10 P-values from TWAS for breast cancer-specific survival in iCOGs (left) and MDD in PGC (right) with local-only models and MOSTWAS models. (PDF) [file pgen.1009398.s009.pdf]

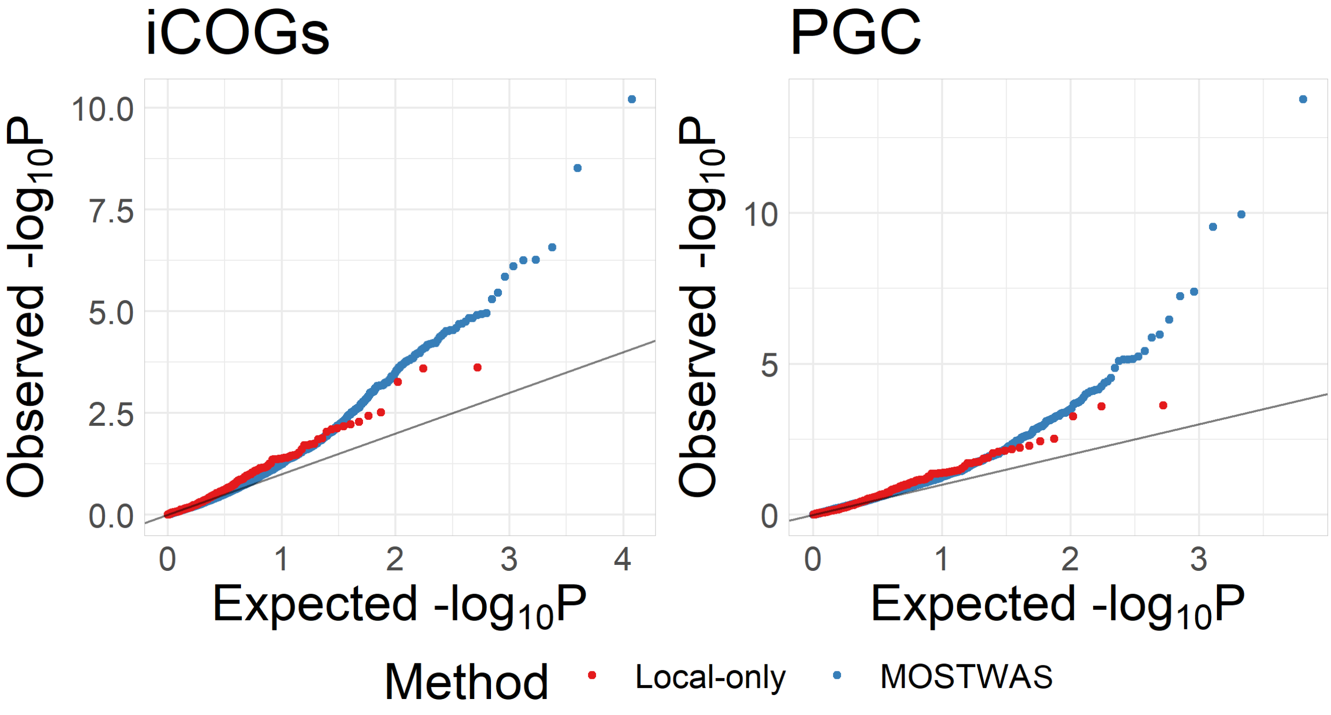

Figure S8: *Comparison of QQ-plots from TWAS associations.* QQ-plots of $-\log_{10}\ P$-values from TWAS for breast cancer-specific survival in iCOGs (left) and MDD in PGC (right) with local-only models and MOSTWAS models.
