# Supplementary material for: MOSTWAS: Multi-Omic Strategies for Transcriptome-Wide Association Studies
Source: PLoS Genet. 2021 Mar 8;17(3):e1009398. doi: 10.1371/journal.pgen.1009398 (PMC7971899; doi:10.1371/journal.pgen.1009398)
Supplement: S9 Fig — Scatterplot of cross-validation adjusted R2 of genes using MOSTWAS (X-axis) and BGW-TWAS (Y-axis) models across 563 samples from TCGA-BRCA. The vertical and horizontal dotted lines provide reference for R2 = 0.01 and the diagonal line is the 45-degree line. Each point is colored blue if the gene has a higher R2 using the MOSTWAS model, gold if the gene has a higher R2 with BGW-TWAS, and grey if the R2 are equal. The proportion of models by method with R2≥0.01 across all imputed genes is provided. (PDF) [file pgen.1009398.s010.pdf]

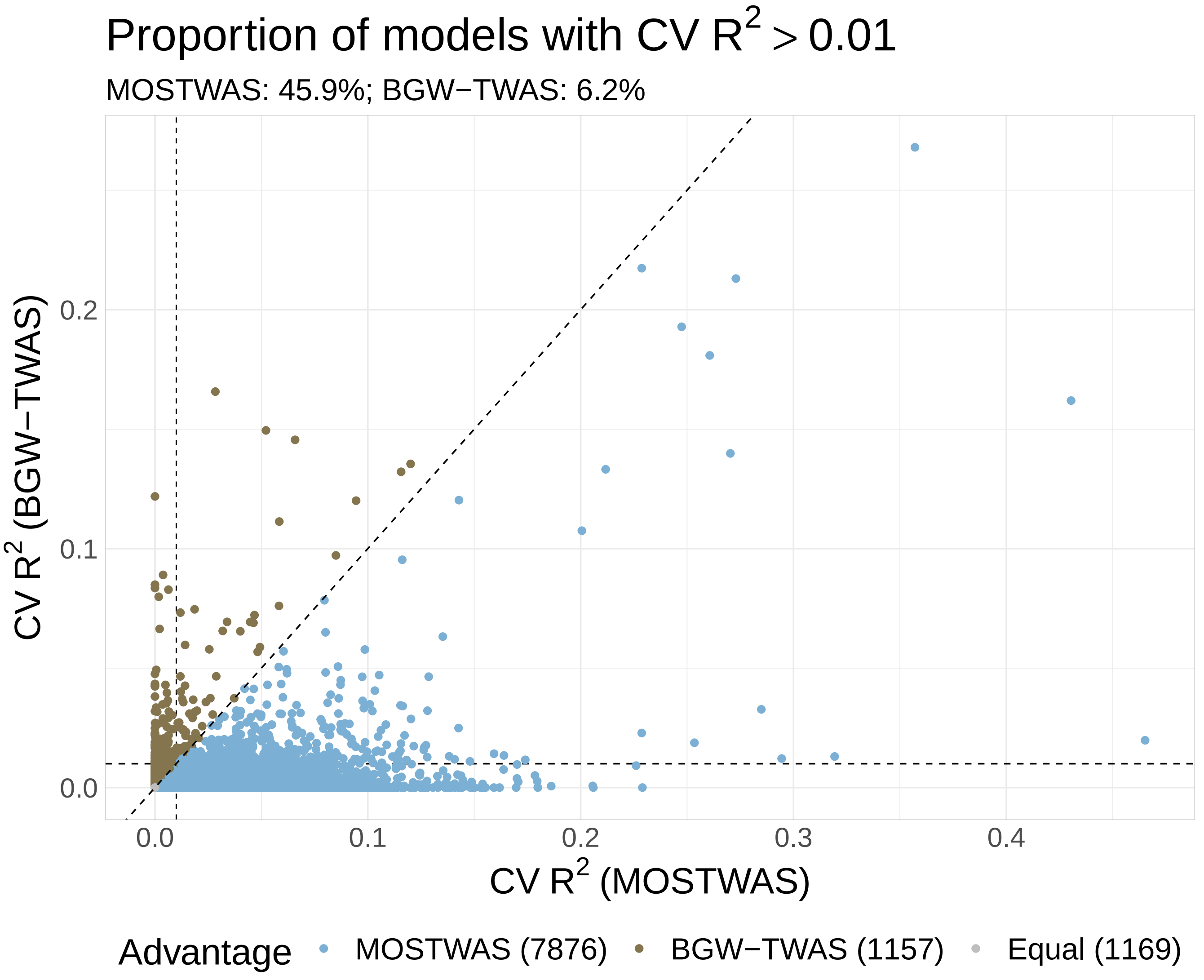

Figure S9: *Comparison of cross-validation R^2 of TCGA models using MOSTWAS and BGW-TWAS.* Scatterplot of cross-validation adjusted R^2 of genes using MOSTWAS (X-axis) and BGW-TWAS (Y-axis) models across 563 samples from TCGA-BRCA. The vertical and horizontal dotted lines provide reference for $R^2 = 0.01$ and the diagonal line is the 45-degree line. Each point is colored blue if the gene has a higher R^2 using the MOSTWAS model, gold if the gene has a higher R^2 with BGW-TWAS, and grey if the R^2 are equal. The proportion of models by method with $R^2 \geq 0.01$ across all imputed genes is provided.
