# Supplementary material for: MOSTWAS: Multi-Omic Strategies for Transcriptome-Wide Association Studies
Source: PLoS Genet. 2021 Mar 8;17(3):e1009398. doi: 10.1371/journal.pgen.1009398 (PMC7971899; doi:10.1371/journal.pgen.1009398)
Supplement: S10 Fig — Manhattan plot of genome-wide eQTL associations by −log10 P -values (Y-axis) and chromosomal position (X-axis) at P<10−3. SNPs included in each predictive model are highlighted in green. CV R2 under each type of MOSTWAS model is provided. (PDF) [file pgen.1009398.s011.pdf]

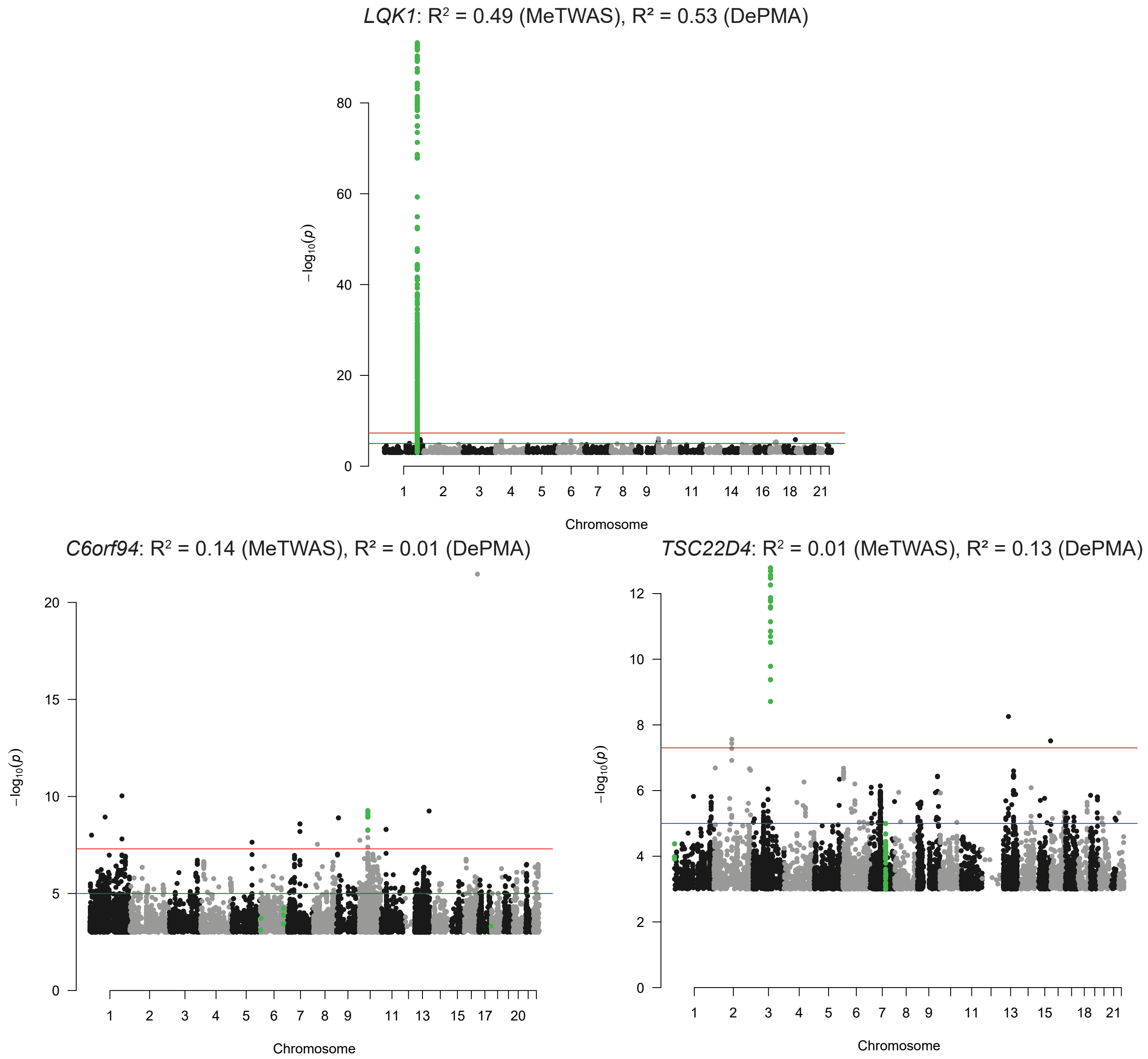

Figure S10: *Comparison of eQTL architectures across LQK1, C6orf94, and TSC22D4.* Manhattan plot of genome-wide eQTL associations by $-log_{10}$ P-values (Y-axis) and chromosomal position (X-axis) at $P < 10^{-3}$. SNPs included in each predictive model are highlighted in green. CV R^2 under each type of MOSTWAS model is provided.
