# Supplementary material for: MOSTWAS: Multi-Omic Strategies for Transcriptome-Wide Association Studies
Source: PLoS Genet. 2021 Mar 8;17(3):e1009398. doi: 10.1371/journal.pgen.1009398 (PMC7971899; doi:10.1371/journal.pgen.1009398)
Supplement: S11 Fig — Mean and standard deviation of per-gene computation time across 50 randomly selected genes in TCGA-BRCA. Computations here were done in serial on a 3.0 GHz processor with 8 gigabytes of RAM. (PDF) [file pgen.1009398.s012.pdf]

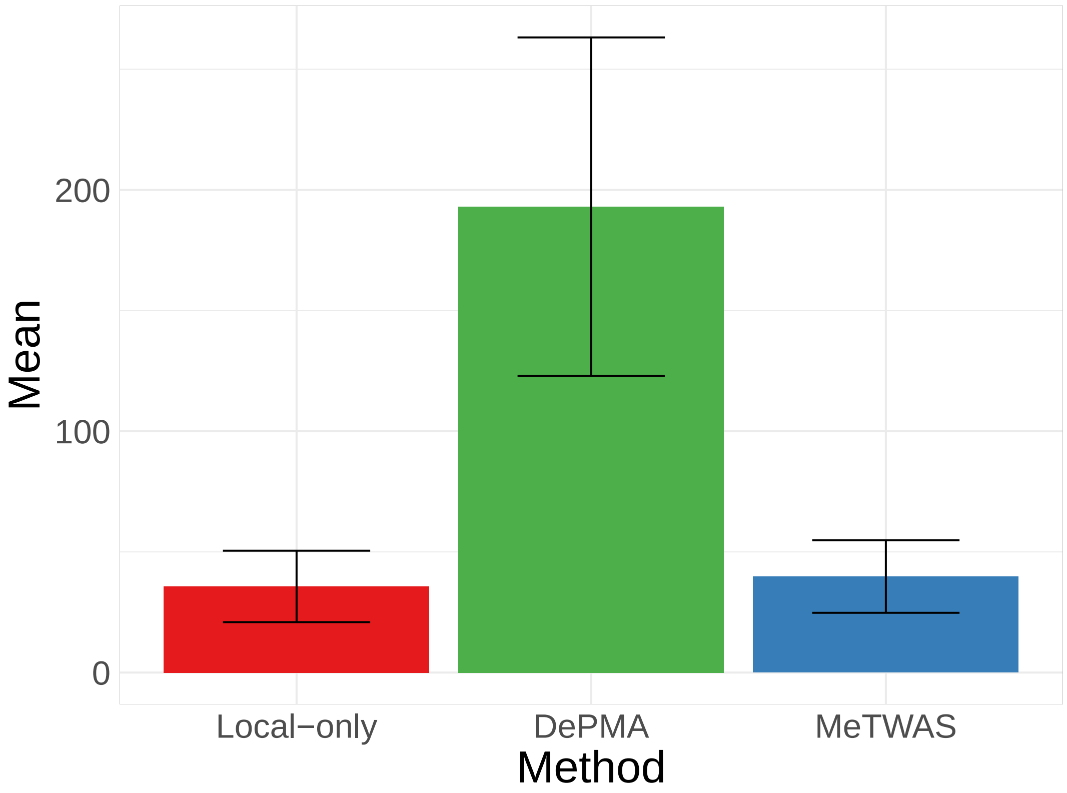

Figure S11: *Comparison of computation times between local-only and MOSTWAS modelling.* Mean and standard deviation of per-gene computation time across 50 randomly selected genes in TCGA-BRCA. Computations here were done in serial on a 3.0 GHz processor with 8 gigabytes of RAM.
