# Supplementary material for: MOSTWAS: Multi-Omic Strategies for Transcriptome-Wide Association Studies
Source: PLoS Genet. 2021 Mar 8;17(3):e1009398. doi: 10.1371/journal.pgen.1009398 (PMC7971899; doi:10.1371/journal.pgen.1009398)
Supplement: S12 Fig — Comparison of predictive performance (Y-axis) of two-step regression (labelled as MeTWAS) and one-step regression (labelled no selection) across 100 simulations across various causal proportions of eQTLs (vertically arranged), local expression heritability (horizontal), and distal expression heritability (X-axis). (PDF) [file pgen.1009398.s013.pdf]

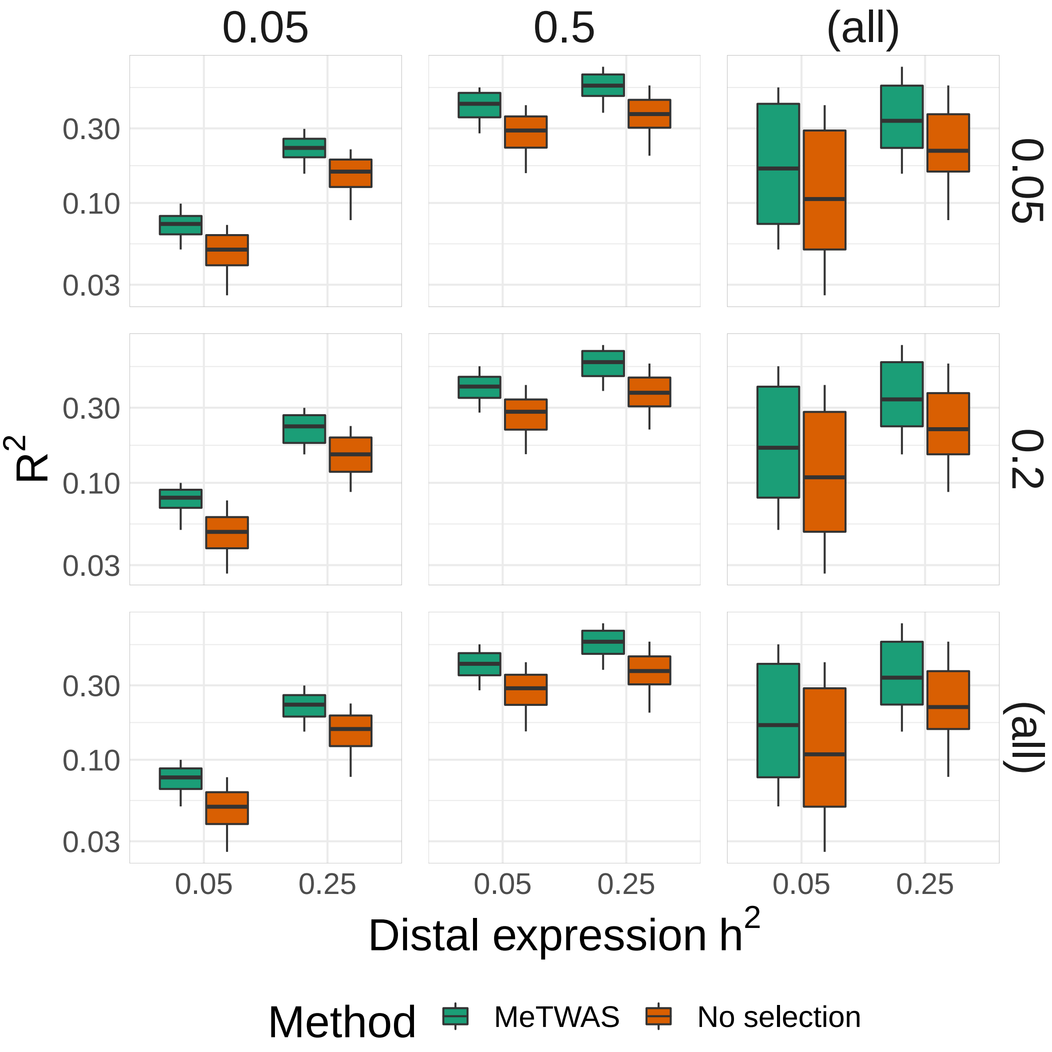

Figure S12: *Comparison of predictive ability of MeTWAS with one- and two-step regression.* Comparison of predictive performance (Y-axis) of two-step regression (labelled as MeTWAS) and one-step regression (labelled no selection) across 100 simulations across various causal proportions of eQTLs (vertically arranged), local expression heritability (horizontal), and distal expression heritability (X-axis).
