# Supplementary material for: MOSTWAS: Multi-Omic Strategies for Transcriptome-Wide Association Studies
Source: PLoS Genet. 2021 Mar 8;17(3):e1009398. doi: 10.1371/journal.pgen.1009398 (PMC7971899; doi:10.1371/journal.pgen.1009398)
Supplement: S13 Fig — Power (Y-axis, left) and computational time (Y-axis, right) to detect a true large absolute total mediation effect and computation speed over various eQTL panel sample sizes (X-axis) in 10,000 simulations of mediation testing triplets. (PDF) [file pgen.1009398.s014.pdf]

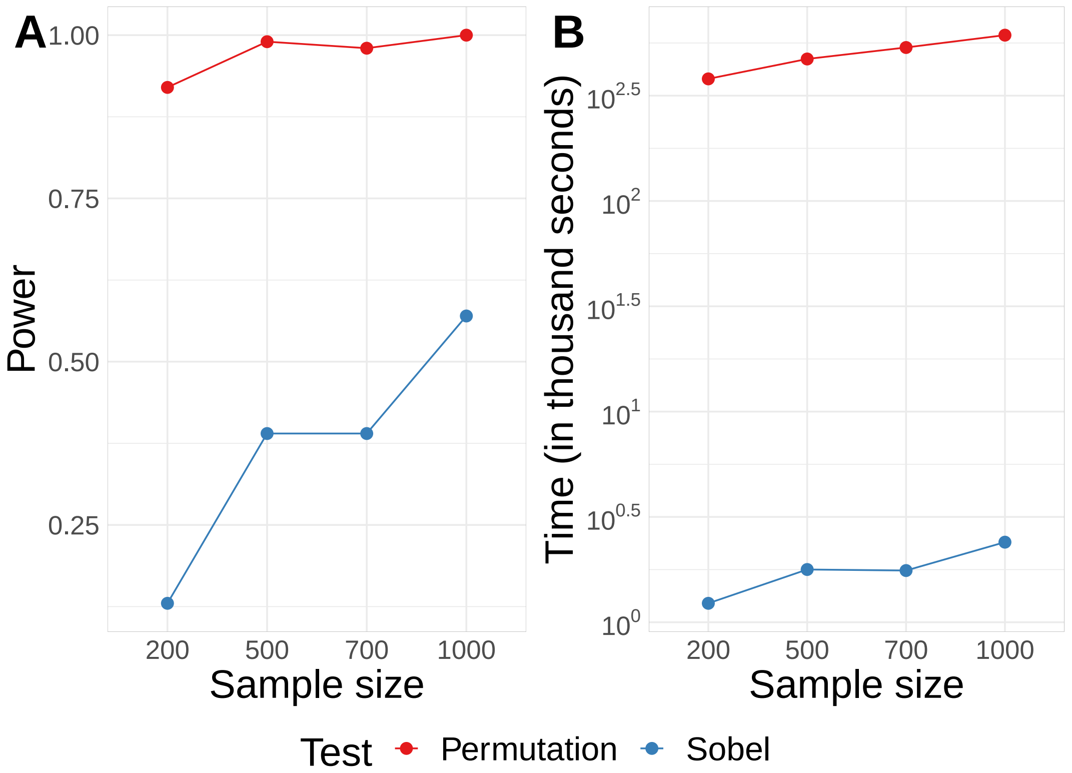

Figure S13: *Comparison of test power and computational speed of Sobel asymptotic and permutation tests of total mediation effect.* Power (Y-axis, left) and computational time (Y-axis, right) to detect a true large absolute total mediation effect and computation speed over various eQTL panel sample sizes (X-axis) in 10,000 simulations of mediation testing triplets.
