# Supplementary material for: MOSTWAS: Multi-Omic Strategies for Transcriptome-Wide Association Studies
Source: PLoS Genet. 2021 Mar 8;17(3):e1009398. doi: 10.1371/journal.pgen.1009398 (PMC7971899; doi:10.1371/journal.pgen.1009398)
Supplement: S1 Table — The mean and standard deviation of h2 across all genes that are significantly heritable with the genetic loci considered in the design matrix of each predictive model. (PDF) [file pgen.1009398.s015.pdf]

MOSTWAS: Multi-omic strategies for transcriptome-wide association studies

Supplemental Tables

	TCGA-BRCA	ROS/MAP
Local-only	0.131 (0.182)	0.077 (0.129)
MeTWAS	0.267 (0.175)	0.146 (0.110)
DePMA	0.401 (0.234)	0.367 (0.132)

Table S1: Comparison of h^2 across local-only, MeTWAS, and DePMA predictive models. The mean and standard deviation of h^2 across all genes that are significantly heritable with the genetic loci considered in the design matrix of each predictive model.
