# Supplementary material for: MOSTWAS: Multi-Omic Strategies for Transcriptome-Wide Association Studies
Source: PLoS Genet. 2021 Mar 8;17(3):e1009398. doi: 10.1371/journal.pgen.1009398 (PMC7971899; doi:10.1371/journal.pgen.1009398)
Supplement: S2 Table — TWAS associations (weighted Z-score and FDR-adjusted P-value) with late-onset Alzheimer’s risk from GWAS statistics from IGAP. The top IGAP GWAS SNP in the identified loci with its location and P-value are provided. For the 6 loci with significant TWAS associations, the FDR-adjusted P-value for the follow-up distal SNP added last test is provided. (PDF) [file pgen.1009398.s016.pdf]

Gene	Z-statistic (FDR-adjusted P)	Cross-validation R^2	Top GWAS location (P-value)	Permutation P-value	Added-last FDR-adjusted P-value
ABCA7	1.36 (0.24)	0.011	Chr19:553066 (0.135)	NA	NA
ADAM10	0.37 (0.72)	0.014	Chr15:59052072 (1.68×10^{-4})	NA	NA
AKAP9	-2.03 (0.08)	0.021	Chr7:92226814 (8.7×10^{4})	9.9×10^{-4}	0.081
APOE	4.47 (1.1×10^{-4})	0.112	Chr7:12268758 (0.028)	9.9×10^{-4}	0.045
BIN1	-0.36 (0.72)	0.010	Chr22:24199787 (8.53×10^{-4})	NA	NA
CD2AP	1.52 (0.20)	0.011	Chr6:47432637 (1.23×10^{-4})	NA	NA
CLU	-2.41 (0.06)	0.012	Chr8:27465312 (1.33×10^{-4})	0.83	NA
FERMT2	2.10 (0.08)	0.012	Chr14:53305626 (0.14)	0.212	NA
NOTCH3	-0.73 (0.59)	0.012	Chr19:15710624 (3.81×10^{-4})	NA	NA
MEF2C	2.15 (0.09)	0.016	Chr5:88359039 (0.020)	0.78	NA
PLCG2	-2.40 (0.06)	0.010	Chr16:81879218 (0.037)	0.66	NA
SORL1	2.91 (0.03)	0.043	Chr11:121446813 (0.032)	0.04	4.57×10^{-3}
TREM2	-0.36 (0.72)	0.011	Chr1:1012483 (0.075)	NA	NA
ZCWPW1	2.02 (0.08)	0.213	Chr7:100435157 (0.075)	0.03	1.31×10^{-5}

Table S2: Summary statistics for known Alzheimer's risk-associated loci identified by MOSTWAS models. TWAS associations (weighted Z-score and FDR-adjusted P-value) with late-onset Alzheimer's risk from GWAS statistics from IGAP. The top IGAP GWAS SNP in the identified loci with its location and P-value are provided. For the 6 loci with significant TWAS associations, the FDR-adjusted P-value for the follow-up distal SNP added last test is provided.
