# Supplementary material for: MOSTWAS: Multi-Omic Strategies for Transcriptome-Wide Association Studies
Source: PLoS Genet. 2021 Mar 8;17(3):e1009398. doi: 10.1371/journal.pgen.1009398 (PMC7971899; doi:10.1371/journal.pgen.1009398)
Supplement: S3 Table — TWAS associations with Alzheimer’s risk from GWAS statistics from IGAP with P<2.5×10^−6^ and permutation P<0.05. The top IGAP GWAS SNP in the identified loci with its location and P-value are provided. (PDF) [file pgen.1009398.s017.pdf]

Gene	TWAS Z (Distal Z)	P-value	Top GWAS SNP (P-value)	Permutation P-value
ANAPC4	-7.53 (NA)	5.02×10^{-14}	4:24878689 (0.01101)	0.00
KIF1B	-7.03 (-9.05)	2.01×10^{-12}	8:134708982 (0.01176)	0.03
SDS	-6.87 (-1.02)	6.24×10^{-12}	1:950243 (0.3102)	0.00
HLA-DRB1	6.8 (-5.03)	1.05×10^{-11}	6:32569659 (0.0001189)	0.02
DDIT3	-6.55 (-2.35)	5.93×10^{-11}	1:768253 (0.1308)	0.00
ACP6	6.08 (NA)	1.20×10^{-9}	1:147301176 (0.0901)	0.00
FAM111A	5.91 (NA)	3.43×10^{-9}	11:60103385 (0.000422)	0.00
TCEB2	-5.88 (-0.11)	4.09×10^{-9}	16:2779236 (0.094)	0.00
OGDH	-5.82 (-7.47)	6.03×10^{-9}	13:43916682 (0.02323)	0.01
CACNG3	5.68 (2.77)	1.34×10^{-8}	19:47154484 (0.001749)	0.01
CADPS2	-5.6 (-4.93)	2.18×10^{-8}	7:121983707 (0.00519)	0.00
CLEC11A	-5.34 (1.18)	9.13×10^{-8}	19:51220996 (0.1121)	0.03
GIF	-5.28 (NA)	1.27×10^{-7}	11:60103385 (0.000422)	0.00
IL23A	5.08 (2.35)	3.77×10^{-7}	1:768253 (0.1308)	0.00
PDE1C	4.88 (8.97)	1.05×10^{-6}	6:157456033 (0.06012)	0.00
APOC1	4.84 (-2.11)	1.30×10^{-6}	1:1000156 (0.01687)	0.01
CCDC8	4.8 (NA)	1.62×10^{-6}	19:45413576 (0.0001009)	0.00
SLC24A6	4.79 (3.63)	1.66×10^{-6}	7:12264297 (0.02613)	0.00

Table S3: Summary statistics for 18 Alzheimer's risk-associated loci identified by MOSTWAS models. TWAS associations with Alzheimer's risk from GWAS statistics from IGAP with $P < 2.5 \times 10^{-}6$ and permutation $P < 0.05$. The top IGAP GWAS SNP in the identified loci with its location and P -value are provided.
