# Supplementary material for: MOSTWAS: Multi-Omic Strategies for Transcriptome-Wide Association Studies
Source: PLoS Genet. 2021 Mar 8;17(3):e1009398. doi: 10.1371/journal.pgen.1009398 (PMC7971899; doi:10.1371/journal.pgen.1009398)
Supplement: S4 Table — TWAS associations with major depressive disorder from GWAS statistics from Psychiatric Genomics Consortium that were replicated with GWAX summary statistics in UK Biobank with permutation test results and added-last Z-statistics with P<2.5×10^−6^ and permutation P<0.05. The top PGC GWAS SNP in the identified loci with its location and P-value are provided. (PDF) [file pgen.1009398.s018.pdf]

Gene	TWAS Z (Distal Z)	P-value	Top GWAS SNP (P-value))	Permutation P-value
RHOF	11.94 (9.43)	7.73×10^{-33}	1:693731 (0.01043)	0
FLAD1	-11.42 (-2.86)	3.50×10^{-30}	1:153990890 (0.0008148)	0.01
ZNF582	9.01 (8.44)	2.04×10^{-19}	6:30074072 (5.127e-09)	0.01
PYGO2	8.49 (3.11)	2.02×10^{-17}	1:154245512 (0.001036)	0
YJEFN3	7.45 (7.28)	9.37×10^{-14}	7:12276011 (5.249e-05)	0.01
KCTD10	7.17 (NA)	7.24×10^{-13}	12:109880996 (0.008794)	0.04
ASL	-6.47 (-6.69)	9.80×10^{-11}	22:29175243 (0.003195)	0.01
GCH1	6.1 (7.92)	1.03×10^{-9}	1:729679 (0.03139)	0.01
USP35	5.85 (7.09)	5.02×10^{-9}	1:2274438 (0.00331)	0.01
IL22RA1	5.39 (NA)	7.20×10^{-8}	1:729679 (0.03139)	0.01
MGP	4.96 (6.26)	7.15×10^{-7}	1:844860 (0.02002)	0.02

Table S4: Summary statistics for 11 MDD risk-associated loci identified by MOSTWAS models. TWAS associations with major depressive disorder from GWAS statistics from Psychiatric Genomics Consortium that were replicated with GWAX summary statistics in UK Biobank with permutation test results and added-last Z-statistics with $P < 2.5 \times 10^{-6}$ and permutation $P < 0.05$. The top PGC GWAS SNP in the identified loci with its location and P-value are provided.
