# Supplementary material for: MOSTWAS: Multi-Omic Strategies for Transcriptome-Wide Association Studies
Source: PLoS Genet. 2021 Mar 8;17(3):e1009398. doi: 10.1371/journal.pgen.1009398 (PMC7971899; doi:10.1371/journal.pgen.1009398)
Supplement: S5 Table — TWAS associations with breast cancer survival from GWAS statistics from iCOGs with permutation test results and added-last Z-statistics with P<2.5×10^−6^ and permutation P<0.05. The top iCOGs GWAS SNP in the identified loci with its location and P-value are provided. (PDF) [file pgen.1009398.s019.pdf]

Gene	TWAS Z (Distal Z)	P-value	Top GWAS SNP (P-value)	Permutation P-value
ATAD1	4.98 (4.62)	6.32×10^{-7}	1:1061794 (0.00310487)	0.00
CTRL	4.92 (3.88)	8.49×10^{-7}	10:2798136 (0.06)	0.01
LOC221710	5.41 (5.08)	6.45×10^{-8}	1:152983865 (0.00085)	0.00
MAP3K6	-4.87 (-4)	1.13×10^{-6}	1:27686314 (0.01)	0.01
TBC1D9	5.23 (4.33)	1.71×10^{-7}	1:961827 (0.00743302)	0.03
TNNC2	-5.27 (-4.48)	1.33×10^{-7}	1:1061794 (0.00310487)	0.00

Table S5: Summary statistics for 6 breast cancer-specific survival-associated loci identified by MOSTWAS models. TWAS associations with breast cancer survival from GWAS statistics from iCOGs with permutation test results and added-last Z-statistics with $P < 2.5 \times 10^{-}6$ and permutation $P < 0.05$. The top iCOGs GWAS SNP in the identified loci with its location and P-value are provided.
