# Supplementary material for: MOSTWAS: Multi-Omic Strategies for Transcriptome-Wide Association Studies
Source: PLoS Genet. 2021 Mar 8;17(3):e1009398. doi: 10.1371/journal.pgen.1009398 (PMC7971899; doi:10.1371/journal.pgen.1009398)
Supplement: Attachment — Submitted filename: Response to Reviewers.docx [file pgen.1009398.s020.docx]

December 11, 2020

Dear Dr. Zhu and Dr. Balding,

Enclosed is a response to reviewers for our manuscript:

*"****MOSTWAS: Multi-Omic Strategies for Transcriptome-Wide Association Studies****"*

We were glad to see that the editors and reviewers found that our newly developed method has merit. Their helpful comments have greatly improved our manuscript. In summary, we have conducted two major new analyses. In summary, these new analyses have resulted in 1 new main figure (original Figure 4 has been split into a new Figure 4 and Figure 5), 1 supplemental table (Supplemental Table S3), and 4 new supplemental figures (new Supplemental Figures S1, S5, S9, and S10) with two supplemental figures removed (original Supplemental Figure S1 is now added to the new Figure 1 and original Supplemental Figure S5 removed entirely).

First, we re-trained MOSTWAS models with a local window of 1 Megabase for consistency with literature and conducted TWAS for the risk of Alzheimer’s disease, major depressive disorder, and breast cancer survival. Increasing this local window resulted in an increase in the number of gene models trained while retaining the 1-2% additive advantage over local-only models in explaining variance in gene expression.

Second, we compared MOSTWAS with a concurrently developed and recently published method that integrates distal variants into TWAS: Bayesian Genome-Wide TWAS (BGW-TWAS) (Luningham *et al* 2020, *AJHG*). In this revision, we have added comparisons between MOSTWAS and BGW-TWAS models in terms of TWAS power through simulation, predictive performance in both ROS/MAP and TCGA-BRCA data, and a comparison of putative risk genes for Alzheimer’s disease detected by MOSTWAS and BGW-TWAS. Our simulation analysis shows generally similar study power between BGW-TWAS and MOSTWAS, but, as we discuss below, we were unable to expand the simulation to fully examine differences between the two methods. We used prefrontal cortex expression data and corresponding genetic data from the PsychENCODE project to impute expression using MOSTWAS and BGW-TWAS models, both trained with ROS/MAP data. Here, we found that, although BGW-TWAS produces more genes, MOSTWAS outperforms BGW-TWAS in 59% of overlapped genes. Similarly, in the TCGA data, more genes pass BGW-TWAS inclusion criteria (~14,000 genes) than with MOSTWAS (~6,000 genes), but in cross-validation, only 6% of these BGW-TWAS models showed $R^{2}>0.01$, while 100% of these MOSTWAS models showed $R^{2}>0.01$, by definition. In external prediction of a hold-out set in TCGA, MOSTWAS outperforms BGW-TWAS in 60% of the overlapped genes. We observed an intersection of 2 genes between sets of TWAS genes detected by the two methods in a study of Alzheimer’s risk. We have added discussion about differences between the methods (e.g., computational cost and inclusion criteria), as well as advantages and disadvantages of each method compared to the other.

We ran into some software bugs and incompatibilities in BGW-TWAS, which we communicated to the authors of Luningham *et al*. We are grateful that the authors addressed these issues by the end of November. These fixes were sufficient to train models in the TCGA-BRCA real data example and add BGW-TWAS into the simulation pipeline presented in the original submission. We were unfortunately unable to benchmark MOSTWAS with BGW-TWAS with more simulation parameters in this revision because of the issues in the code but also the computational cost of BGW-TWAS (see Discussion of Luningham *et al* 2020, *AJHG*). Nevertheless, we believe we have provided a fair comparison of both methods, which provides context about strengths and weaknesses of MOSTWAS in comparison to BGW-TWAS.

In addition, we have added an exploration of differences in eQTL architecture between genes well-predicted by MeTWAS or DePMA but not the other. During the revision process, we have also modified the MOSTWAS implementation by using the bigsnpr package in R, allowing us to use more efficient memory mapping and considerably reduce overall computational time.

We believe that this revision has greatly improved our manuscript and thank the editors and reviewers for their helpful feedback.

| Arjun Bhattacharya  Postdoctoral Fellow  Department of Pathology and Laboratory Medicine  University of California, Los Angeles | Michael I. Love  Assistant Professor  Department of Biostatistics  Department of Genetics  University of North Carolina at Chapel Hill |
| --- | --- |

Below is a point-by-point response to Reviewer 1 for our manuscript:

*"****MOSTWAS: Multi-Omic Strategies for Transcriptome-Wide Association Studies****"*

**SUMMARY OF REVISIONS**

We were glad to see that reviewer found that our newly developed method has merit. Their helpful comments have greatly improved our manuscript. In summary, we have conducted two major new analyses. First, we re-trained MOSTWAS models with a 1 Megabase local window and conducted a TWAS for risk of Alzheimer’s disease, major depressive disorder, and breast cancer survival using these newly trained models. Second, we conducted a comparison of MOSTWAS and BGW-TWAS models using the simulations and real data examples presented in the original submission. These new analyses have resulted in 1 new main figure (original Figure 4 has been split into a new Figure 4 and Figure 5), 1 supplemental table (Supplemental Table S3), and 4 new supplemental figures (new Supplemental Figures S1, S5, S9, and S10) with two supplemental figures removed (original Supplemental Figure S1 is now added to the new Figure 1 and original Supplemental Figure S5 removed entirely).

During the revision process, we have also modified the MOSTWAS implementation by using the bigsnpr package in R, allowing us to use more efficient memory mapping and considerably reduce overall computational time.

We believe that this revision has greatly improved our manuscript and thank the reviewer for their helpful feedback.

**POINT-BY-POINT RESPONSES**

Reviewer comments are bolded, and responses from the authors are indented.

**Reviewer #1: Bhattacharya et al. developed two novel TWAS (Transcriptome-Wide Association Studies) methods to incorporate multi-omics data: mediator-enriched TWAS (MeTWAS) and distal-eQTL prioritization via mediation analysis (DePMA). As compared to most existing TWAS methods that only relies on local-eQTLs, the two proposed methods borrow additional information from distal-eQTLs and other omics data such as DNA methylation and microRNA. Both methods adopt rigorous mediation analysis approaches. Below I list some minor issues that may further improve the manuscript.**

**1. Incorporating omics data in TWAS has become a hot research topic recently. For instance, the following related paper, initially been posted on bioRxiv on March 6, also considers distal-eQTLs in TWAS. “Bayesian Genome-wide TWAS method to leverage both cis- and trans-eQTL information through summary statistics”** [**https://www.biorxiv.org/content/10.1101/2020.03.05.979187v2**](https://www.biorxiv.org/content/10.1101/2020.03.05.979187v2) **It is worth discussion if a comparison is too much work. It is especially key for reproducibility across methods. A quick look does not find overlap genes for Alzheimer’s disease.**

The reviewer makes a good point here – Bayesian Genome-Wide TWAS (BGW-TWAS) is a concurrent TWAS method that includes distal variants in TWAS that was preprinted within a month of our original preprint. We added BGW-TWAS into our simulation analysis, compared predictive performance of MOSTWAS against that of BGW-TWAS (Luningham *et al* 2020, *AJHG*) in both real data examples, and added a comparison of putative risk genes for Alzheimer’s disease detected by MOSTWAS and BGW-TWAS. We used prefrontal cortex expression data and corresponding genetic data from the PsychENCODE project to impute expression using MOSTWAS and BGW-TWAS models, both trained with ROS/MAP data. Here, we found that, although BGW-TWAS produces more genes, MOSTWAS outperforms BGW-TWAS in 59% of overlapped genes. Similarly, in the TCGA data, more genes pass BGW-TWAS inclusion criteria (~14,000 genes) than with MOSTWAS (~6,000 genes), but in cross-validation, only 6% of these BGW-TWAS models showed $R^{2}>0.01$, where 100% of these MOSTWAS models showed $R^{2}>0.01$, by definition. In external prediction of a hold-out set in TCGA, MOSTWAS outperforms BGW-TWAS in 60% of the overlapped genes. We also find an intersection of 2 genes between sets of TWAS genes detected by the two methods. We have added discussion about differences between the methods (e.g., computational cost and inclusion criteria), as well as advantages and disadvantages of each method compared to the other. Our comparison of these methods using ROS/MAP models provides some context about strengths and weaknesses of MOSTWAS in comparison.

In the Results section, we add the following about the comparison in predictive performances:

“We also compared MOSTWAS external predictive performance with BGW-TWAS, which employs Bayesian variable selection to train predictive models using both local- and distal-eQTLs [30]. Here, as BGW-TWAS models were trained on the entire ROS/MAP dataset, we extracted expression data from the PsychENCODE project for 646 samples from dorsolateral prefrontal cortex tissue and corresponding individual-level genotype data; these 646 samples are derived from patients in the control group, with no symptoms of schizophrenia or bipolar disorder [38,39]. We then imputed expression using both MOSTWAS and BGW-TWAS models trained using ROS/MAP and assessed predictive performance via McNemar’s adjusted $R^{2}$. Here, we note that BGW-TWAS uses a different inclusion criteria that MOSTWAS, where a BGW-TWAS model is trained if at least one eQTL with non-zero effect and sufficiently large posterior causal probability is detected without heritability estimation or cross-validation performance [30]. Possibly due to MOSTWAS’s more conservative inclusion criteria, BGW-TWAS imputes a larger number of genes than MOSTWAS (11,990 versus 4,931, respectively). However, of the 3,385 genes that both MOSTWAS and BGW-TWAS imputed, MOSTWAS models generally have larger adjusted $R^{2}$ in the PsychENCODE data, where 59% of genes have higher $R^{2}$ with MOSTWAS compared to BGW-TWAS (**Figure 4B**). Across all genes imputable by each method, MOSTWAS and BGW-TWAS have roughly the same proportion of predicted at external validation $R^{2}\geq0.01$ (MOSTWAS 13.6%, BGW-TWAS 14.3%).”

Also, in the Results, we add the following about the comparison in Alzheimer’s risk TWAS genes:

“We also assessed TWAS associations for Alzheimer’s disease risk using MOSTWAS for the 11 autosomal risk genes identified by Luningham et al using BGW-TWAS [37]. Due to more conservative inclusion criteria in MOSTWAS, we only trained significant models for 5 of these genes (*AEP1*, *BTN3A2*, *GPX1*, *APOC1*, and *HLA-DRB1*). At $P <2.5\times{10}^{-6}$, we found significant associations with risk of Alzheimer’s disease for *APOC1* ($Z = 4.84, P = 1.29\times{10}^{-6}$) and *HLA-DRB1* ($Z = 6.80, P = 1.05\times{10}^{-11}$) that also passed permutation testing. Overall, across all 5407 genes with significant MOSTWAS models, 18 genes showed TWAS associations at $P<2.5\times{10}^{-6}$ and passed permutation testing at nominal $P<0.05$ (**Figure 5B**, **Supplemental Table S3, Supplemental Figure S4**).”

In the Results section about the TCGA-BRCA example, we add:

“We also trained expression models with BGW-TWAS using TCGA-BRCA data, computed cross-validation adjusted $R^{2}$ across three folds, and imputed expression into this external dataset for comparison. We trained 14,029 models using BGW-TWAS at default input parameters with at least one SNP in the model. Over 3-fold cross-validation, we found that 6.2% of these models met $R^{2}>0.01$. Comparing the 10,202 genes with BGW-TWAS models and $h^{2}>0$ with $P<0.05$, as calculated in the MOSTWAS pipeline, we found that 77% of MOSTWAS models outperform BGW-TWAS models in cross-validation (**Supplemental Figure S9**). Of the 4,682 genes that passed both MOSTWAS and BGW-TWAS inclusion criteria, MOSTWAS models generally have larger adjusted $R^{2}$ in the external dataset, where nearly 60% of genes have higher $R^{2}$ with MOSTWAS compared to BGW-TWAS (**Figure 4C**). Across all genes with models of each method, MOSTWAS (5,897 significant models) trained more models with external validation $R^{2}\geq0.01$ (MOSTWAS 46.9%, BGW-TWAS 38.5%).”

Lastly, we add in Discussion some context about the strengths and weaknesses of each method:

“We compared MOSTWAS to a contemporary TWAS method that incorporates distal-eQTLs, BGW-TWAS [30]. BGW-TWAS can impute a larger number of gene models than MOSTWAS, but among genes both BGW-TWAS and MOSTWAS were able to impute in external PsychENCODE and TCGA-BRCA data [38,39], MOSTWAS generally showed larger predictive adjusted $R^{2}$. MOSTWAS’s stricter inclusion criterion (thresholds for both expression heritability and cross-validation performance) may lead to fewer genes trained and ultimately tested; for example, when we subjected BGW-TWAS models trained with TCGA-BRCA data to cross-validation, few met MOSTWAS’s inclusion criterion of cross-validation adjusted $R^{2}>0.01$. We believe that assessing expression heritability signal and cross-validating models prior to TWAS is necessary: as Ding *et al* points out in their systematic power analysis, expression heritability is a major factor in determining TWAS power [82]. In a TWAS for Alzheimer’s disease, results from MOSTWAS and BGW-TWAS showed two overlap genes, out of a total of 18 and 13 putative TWAS genes in each respective method. BGW-TWAS employs Bayesian methodology to scan the entire genome for potentially causal distal-eQTLs and can build models for more genes at a large computational cost [30]. For example, BGW-TWAS requires approximately 30 minutes per gene with 3 GB of memory with parallel computation over 4 cores, whereas DePMA (the slower of the two MOSTWAS methods) takes approximately 6 minutes per gene with 8 GB of memory with serial implementation. BGW-TWAS only requires genetic and transcriptomic data for its prediction, an advantage over MOSTWAS. On the other hand, MOSTWAS has an interpretational advantage over BGW-TWAS by pinning distal variants to mediating biomarkers and testing trait association at these distal loci with the added-last test.”

**2. Which local-only methods were used for comparison in simulations and data analysis? Given different local-only algorithms, it would be precise to explicitly note what exact method was used each time when local-only models were mentioned.**

This is an important note from the reviewer. Local-only models here refer to an implementation of FUSION without Bayesian sparse linear mixed modelling (Gusev *et al* 2016, *Nature Genetics*). We state in Results now:

“We use FUSION with Bayesian sparse linear mixed modelling (BSLMM) as the comparison local-only modelling implementation throughout all analyses, unless otherwise noted [4]...”

Throughout the Results sections, we also point to “FUSION without BSLMM” to reinforce to the reader that this was our choice for the overwhelming majority of local-only modeling. In the Results section, when we present comparisons of recapitulation of GWAS signals in Alzheimer’s disease risk, we now write:

“We next conducted association tests for known Alzheimer’s disease risk loci using two local-only methods (PrediXcan [3] and TIGAR [36]) and the best MOSTWAS model (selected by comparing MeTWAS and DePMA cross-validation R^2^) trained in ROS/MAP and summary-level GWAS data from the International Genomics of Alzheimer’s Project (IGAP) [37]. This comparison is similar to an analysis of recapitulation of GWAS signals in Alzheimer’s disease from Nagpal et al [36]. “

In Methods section “”Model training and association testing in ROS/MAP and TCGA-BRCA”, we now state:

“Local-only modeling, unless otherwise noted, is a default implementation of FUSION without considering Bayesian sparse linear mixed modelling (BSLMM), as BSLMM is computational expensive [4]. We consider default PrediXcan and DPR modeling only in the comparison of recapitulated GWAS signals in Alzheimer’s disease risk [3,36].”

**3. The authors defined distal-eQTL with a distance of 0.5 Mb, while many other studies used a distance of 1 Mb. I am wondering if this would affect the performance of the proposed TWAS methods and biological interpretation.**

The reviewer is correct in pointing this out: we have updated the real data analysis to reflect the traditional definition of a local window of 1 Mb. This greatly improves predictive performance of both local-only and MOSTWAS models mainly by increasing the number of significant models (heritability greater than 0 with nominal P < 0.05 and CV R^2^ > 0.01). Using the ROSMAP dataset, using a local window of 1 Mb, we now have 1,385, 2,287, and 4,725 significant local-only, MeTWAS, and DePMA models, respectively, compared to 267, 911, and 2,934, previously with a local window of 0.5 Mb. Using the TCGA-BRCA dataset, we now have 849, 2,641, and 4,862 significant local-only, MeTWAS, and DePMA models, respectively, compared to 280, 709, and 1,185, previously with a local window of 0.5 Mb. Median R^2^ of MeTWAS and DePMA retain their 1-2% additive advantage in percent variance of expression explained over local-only models, as we write in the Abstract. We update our real data results with these new models throughout the paper. This increase of the local window also aided in the replication rate of both local-only and MOSTWAS associations.

**4. Some figures need improvement.**

**Fig 3, making each subfigure square would facilitate reading;**

**Fig 4 AB, no legends for the three colors. What do P and D mean in Fig 4B?**

Thank you for pointing this out about the figures. We have squared each panel of Figure 3 and added legends for these subfigures of the original Figure 4. The P and D are labeled in the caption for the original Figure 4B. Specifically, we write in the caption:

“Loci are labeled with P if the permutation test achieves FDR-adjusted P < 0.05 and D if the added-last test achieves FDR-adjusted P < 0.05.”

**5. There are some typos.**

**Line 70, “many distal-eQTLs are often eQTLs for one of more of their local genes”**

**“one of more of” should be “one or more of”?**

Thank you for pointing this typo out. We have fixed it.

**In the section of “real data applications in brain tissue,” “tumor” appeared multiple times. It should be avoided because the data are about Alzheimer’s disease.**

Thank you for pointing this error out. We have fixed it.

**Line 400, typo “sties”.**

Thank you for pointing this typo out. We have fixed it.

**Line 472, redundant words, “we considered two scenarios.”**

Thank you for pointing this typo out. We have fixed it.

**Supplemental Methods, formula (2), the second equation, \beta_M should be w_G?**

Thank you for pointing this error out. We have fixed it.

Below is a point-by-point response to Reviewer 2 for our manuscript:

*"****MOSTWAS: Multi-Omic Strategies for Transcriptome-Wide Association Studies****"*

**SUMMARY OF REVISIONS**

We were glad to see that reviewer found that our newly developed method has merit. Their helpful comments have greatly improved our manuscript. In summary, we have conducted two major new analyses. First, we re-trained MOSTWAS models with a 1 Megabase local window and conducted a TWAS for risk of Alzheimer’s disease, major depressive disorder, and breast cancer survival using these newly trained models. Second, we conducted a comparison of MOSTWAS and BGW-TWAS models using the simulations and real data examples presented in the original submission. These new analyses have resulted in 1 new main figure (original Figure 4 has been split into a new Figure 4 and Figure 5), 1 supplemental table (Supplemental Table S3), and 4 new supplemental figures (new Supplemental Figures S1, S5, S9, and S10) with two supplemental figures removed (original Supplemental Figure S1 is now added to the new Figure 1 and original Supplemental Figure S5 removed entirely).

During the revision process, we have also modified the MOSTWAS implementation by using the bigsnpr package in R, allowing us to use more efficient memory mapping and considerably reduce overall computational time.

We believe that this revision has greatly improved our manuscript and thank the reviewer for their helpful feedback.

**POINT-BY-POINT RESPONSES**

Reviewer comments are bolded, and responses from the authors are indented.

**Reviewer #2: The authors proposed two approaches to consider potential distal-eQTL for improving the prediction accuracy of gene expression and then the TWAS power. Here are my comments:**

**1. Although it is good to see that additional distal-eQTL could play an important role in TWAS, the authors did not describe their methods clearly in the main text. I think it would be helpful to include brief formulas to introduce the models of these two proposed methods in the main text. Include Figure S1 as part of Figure 1 would also help.**

This is a good point. We tried to limit the mathematical notation in the text to allow for readability for a diverse audience. We have included the model scheme from Supplemental Figure S1 to the main Figure 1.

**2. The authors shall also compare with the recently published BGW-TWAS method (**[**https://doi.org/10.1016/j.ajhg.2020.08.022**](https://doi.org/10.1016/j.ajhg.2020.08.022)**) that considers both cis- and trans- eQTL for TWAS and used the same ROS/MAP application data set.**

The reviewer makes a great suggestion here. We added BGW-TWAS into our simulation analysis, compared predictive performance of MOSTWAS against that of BGW-TWAS (Luningham *et al* 2020, *AJHG*) in both real data examples, and added a comparison of putative risk genes for Alzheimer’s disease detected by MOSTWAS and BGW-TWAS. We used prefrontal cortex expression data and corresponding genetic data from the PsychENCODE project to impute expression using MOSTWAS and BGW-TWAS models, both trained with ROS/MAP data. Here, we found that, although BGW-TWAS produces more genes, MOSTWAS outperforms BGW-TWAS in 59% of overlapped genes. Similarly, in the TCGA data, more genes pass BGW-TWAS inclusion criteria (~14,000 genes) than with MOSTWAS (~6,000 genes), but in cross-validation, only 6% of these BGW-TWAS models showed $R^{2}>0.01$, where 100% of these MOSTWAS models showed $R^{2}>0.01$, by definition. In external prediction of a hold-out set in TCGA, MOSTWAS outperforms BGW-TWAS in 60% of the overlapped genes. We also find an intersection of 2 genes between sets of TWAS genes detected by the two methods. We have added discussion about differences between the methods (e.g., computational cost and inclusion criteria), as well as advantages and disadvantages of each method compared to the other. Our comparison of these methods using ROS/MAP models provides some context about strengths and weaknesses of MOSTWAS in comparison.

In the Results section, we add the following about the comparison in predictive performances:

“We also compared MOSTWAS external predictive performance with BGW-TWAS, which employs Bayesian variable selection to train predictive models using both local- and distal-eQTLs [30]. Here, as BGW-TWAS models were trained on the entire ROS/MAP dataset, we extracted expression data from the PsychENCODE project for 646 samples from dorsolateral prefrontal cortex tissue and corresponding individual-level genotype data; these 646 samples are derived from patients in the control group, with no symptoms of schizophrenia or bipolar disorder [38,39]. We then imputed expression using both MOSTWAS and BGW-TWAS models trained using ROS/MAP and assessed predictive performance via McNemar’s adjusted $R^{2}$. Here, we note that BGW-TWAS uses a different inclusion criteria that MOSTWAS, where a BGW-TWAS model is trained if at least one eQTL with non-zero effect and sufficiently large posterior causal probability is detected without heritability estimation or cross-validation performance [30]. Possibly due to MOSTWAS’s more conservative inclusion criteria, BGW-TWAS imputes a larger number of genes than MOSTWAS (11,990 versus 4,931, respectively). However, of the 3,385 genes that both MOSTWAS and BGW-TWAS imputed, MOSTWAS models generally have larger adjusted $R^{2}$ in the PsychENCODE data, where 59% of genes have higher $R^{2}$ with MOSTWAS compared to BGW-TWAS (**Figure 4B**). Across all genes imputable by each method, MOSTWAS and BGW-TWAS have roughly the same proportion of predicted at external validation $R^{2}\geq0.01$ (MOSTWAS 13.6%, BGW-TWAS 14.3%).”

Also, in the Results, we add the following about the comparison in Alzheimer’s risk TWAS genes:

“We also assessed TWAS associations for Alzheimer’s disease risk using MOSTWAS for the 11 autosomal risk genes identified by Luningham et al using BGW-TWAS [37]. Due to more conservative inclusion criteria in MOSTWAS, we only trained significant models for 5 of these genes (*AEP1*, *BTN3A2*, *GPX1*, *APOC1*, and *HLA-DRB1*). At $P <2.5\times{10}^{-6}$, we found significant associations with risk of Alzheimer’s disease for *APOC1* ($Z = 4.84, P = 1.29\times{10}^{-6}$) and *HLA-DRB1* ($Z = 6.80, P = 1.05\times{10}^{-11}$) that also passed permutation testing. Overall, across all 5407 genes with significant MOSTWAS models, 18 genes showed TWAS associations at $P<2.5\times{10}^{-6}$ and passed permutation testing at nominal $P<0.05$ (**Figure 5B**, **Supplemental Table S3, Supplemental Figure S4**).”

In the Results section about the TCGA-BRCA example, we add:

“We also trained expression models with BGW-TWAS using TCGA-BRCA data, computed cross-validation adjusted $R^{2}$ across three folds, and imputed expression into this external dataset for comparison. We trained 14,029 models using BGW-TWAS at default input parameters with at least one SNP in the model. Over 3-fold cross-validation, we found that 6.2% of these models met $R^{2}>0.01$. Comparing the 10,202 genes with BGW-TWAS models and $h^{2}>0$ with $P<0.05$, as calculated in the MOSTWAS pipeline, we found that 77% of MOSTWAS models outperform BGW-TWAS models in cross-validation (**Supplemental Figure S9**). Of the 4,682 genes that passed both MOSTWAS and BGW-TWAS inclusion criteria, MOSTWAS models generally have larger adjusted $R^{2}$ in the external dataset, where nearly 60% of genes have higher $R^{2}$ with MOSTWAS compared to BGW-TWAS (**Figure 4C**). Across all genes with models of each method, MOSTWAS (5,897 significant models) trained more models with external validation $R^{2}\geq0.01$ (MOSTWAS 46.9%, BGW-TWAS 38.5%).”

Lastly, we add in Discussion some context about the strengths and weaknesses of each method:

“We compared MOSTWAS to a contemporary TWAS method that incorporates distal-eQTLs, BGW-TWAS [30]. BGW-TWAS can impute a larger number of gene models than MOSTWAS, but among genes both BGW-TWAS and MOSTWAS were able to impute in external PsychENCODE and TCGA-BRCA data [38,39], MOSTWAS generally showed larger predictive adjusted $R^{2}$. MOSTWAS’s stricter inclusion criterion (thresholds for both expression heritability and cross-validation performance) may lead to fewer genes trained and ultimately tested; for example, when we subjected BGW-TWAS models trained with TCGA-BRCA data to cross-validation, few met MOSTWAS’s inclusion criterion of cross-validation adjusted $R^{2}>0.01$. We believe that assessing expression heritability signal and cross-validating models prior to TWAS is necessary: as Ding *et al* points out in their systematic power analysis, expression heritability is a major factor in determining TWAS power [82]. In a TWAS for Alzheimer’s disease, results from MOSTWAS and BGW-TWAS showed two overlap genes, out of a total of 18 and 13 putative TWAS genes in each respective method. BGW-TWAS employs Bayesian methodology to scan the entire genome for potentially causal distal-eQTLs and can build models for more genes at a large computational cost [30]. For example, BGW-TWAS requires approximately 30 minutes per gene with 3 GB of memory with parallel computation over 4 cores, whereas DePMA (the slower of the two MOSTWAS methods) takes approximately 6 minutes per gene with 8 GB of memory with serial implementation. BGW-TWAS only requires genetic and transcriptomic data for its prediction, an advantage over MOSTWAS. On the other hand, MOSTWAS has an interpretational advantage over BGW-TWAS by pinning distal variants to mediating biomarkers and testing trait association at these distal loci with the added-last test.”

**3. It is not clearly stated about the number of mediators of different omics- types that were considered in the application studies. For the gene expression trait of each gene, will all mediators available for the study cohort be considered by MeTWAS and DePMA?**

The number of mediators per omic per dataset is provided in the Methods section. In the Methods section “Multi-omic data from ROS/MAP”, we write:

“We intersected to the subset of samples assayed for genotype (at 4,141,537 variants), RNA-seq (15,857 genes), miRNA-seq (247 miRNAs), and DNA methylation (391,626 CpG sites)...”

In the Methods section “Multi-omic data from TCGA-BRCA”, we write:

“We intersected to the subset of samples assayed for genotype (4,564,962 variants), RNA-seq (15,568 genes), miRNA-seq (1,046 miRNAs), and DNA methylation (485,578 CpG sites)...”

For MeTWAS and DePMA, all genes coding for transcription factors, miRNAs, and DNA methylation sites were considered as potential mediators, with QTL detection prior to predictive modeling serving as feature selection. For example, the default for MeTWAS is the 5 mediators (any of the omics) with smallest P-values of association with the gene of interest. The default for DePMA is any distal-eQTL that is a part of mediation test triplet, where a test triplet consists of (1) the gene of interest, (2) a distal-eSNP associated with the expression of the gene (P < 10^-6^), and (3) a set of mediating biomarkers local to and associated with the distal-eSNP (FDR-adjusted P < 0.05).

**4. For the computation time mentioned in the main text, it is not clear whether additional computation time about generating standard eQTL p-values, or identifying associated mediators are included or not.**

These computational times do not include the QTL fitting (both mediators-gene and SNPs-gene) that are necessary for both MOSTWAS methods. We believe that any thorough TWAS requires assessment of eQTL architecture prior to predictive modelling, and thus this eQTL detection step is (or should be) shared across all TWAS methods. We have stated this in Results section, with a brief discussion of computational bottlenecks:

“These computation times do not include QTL detection steps (detecting distal mediators in MeTWAS and distal-eQTLs in DePMA) prior to fitting the predictive models. The computational bottlenecks for MeTWAS and DePMA are fitting mediator prediction models and testing the mediation effect through permutation, respectively.”

In this revision, we have also reworked the implementation to use efficient memory mapping and faster modeling using the bigsnpr package (Privé *et al* 2018, *Bioinformatics*). As evidenced in the new Supplemental Figure S8, this new implementation substantially decreases the fitting time for both models and mitigates a disadvantage of MOSTWAS.

**5. The authors need to be specific about which local-only models were compared in the application studies, as PrediXcan, Fusion, and TIGAR use different statistical models and perform differently.**

We have added specificity here. On the large part, local-only modeling refers to an implementation of FUSION without Bayesian sparse linear mixed modeling. We have clarified this in the Results and Methods sections. We state in Results now:

“We use FUSION with Bayesian sparse linear mixed modelling (BSLMM) as the comparison local-only modelling implementation throughout all analyses, unless otherwise noted [4]...”

Throughout the Results sections, we also point to “FUSION without BSLMM” to reinforce to the reader that this was our choice for the overwhelming majority of local-only modeling. In the Results section, when we present comparisons of recapitulation of GWAS signals in Alzheimer’s disease risk, we now write:

“We next conducted association tests for known Alzheimer’s disease risk loci using two local-only methods (PrediXcan [3] and TIGAR [36]) and the best MOSTWAS model (selected by comparing MeTWAS and DePMA cross-validation R^2^) trained in ROS/MAP and summary-level GWAS data from the International Genomics of Alzheimer’s Project (IGAP) [37]. This comparison is similar to an analysis of recapitulation of GWAS signals in Alzheimer’s disease from Nagpal et al [36]. “

In Methods section “Model training and association testing in ROS/MAP and TCGA-BRCA”, we now state:

“Local-only modeling, unless otherwise noted, is a default implementation of FUSION without considering Bayesian sparse linear mixed modelling (BSLMM), as BSLMM is computational expensive [4]. We consider default PrediXcan and DPR modeling only in the comparison of recapitulated GWAS signals in Alzheimer’s disease risk [3,36].”

**6. In the application study of using ROS/MAP data, as mentioned from lines 199-201, does it mean only 267 genes has training R2 > 0.01 by local-only models, 911 by MeTWAS, and 2934 by DePMA? The number of genes is much less than reported in the TIGAR paper.**

You are correct - those are the numbers of genes that we were able to predict at cross-validation R^2^ > 0.01 in our sample. First, we only consider genes with positive heritability at nominal P < 0.05 for the likelihood ratio test from GCTA GREML analysis, which further reduces the number of genes for which we build models. There is no explicit mention of pre-selecting genes with significantly positive heritability in Nagpal *et al*. In fact, Ding *et al* (Ding et al 2020, *bioRxiv*), in their systematic power analysis of TWAS, show that low expression heritability often leads to misapplication of TWAS methods; we believe, now with substantial evidence from Ding *et al*, that a first step to eliminate genes with low expression heritability is necessary. Second, Nagpal *et al* state in their TIGAR paper:

“If the training model is null, we take the imputation R^2^ as 0 and take the average imputation R^2^ across all 5-fold test samples as 5-fold CV R^2^.”

There is a difference in how Nagpal *et al* and we compute cross-validation R^2^. Whereas Nagpal *et al* takes the average of per-fold R^2^, we collect the cross-validated predictions and compute an adjusted R^2^ between the predicted and observed expression using Theil’s formula, which gives an approximate correction for sample size. Our approach is similar to how Gusev *et al*’s FUSION method calculates heritability and cross-validation R^2^; this calculation leads to more conservative estimates of R^2^.

To show how these differences in the method of calculating R^2^ affects the performance metric, consider this following simple simulation. We generate two vectors $a, b\sim N\left( 0,1 \right)$, where $a, b$ both have length 500. We then scale the last 100 elements of $a$ to be highly correlated with $b$, specifically $a\left[ 401:500 \right]=b\left[ 401:500 \right]+N\left( 0,1 \right).$ Each 100 elements of $a$ and $b$ represents an approximate training-testing fold. We then calculate R^2^ between $a$ and $b$ by calculating per-fold R^2^ and averaging these 5 values (red), taking the squared Pearson correlation between $a$ and $b$ (blue), and taking the McNemar’s adjusted R^2^. As the violin plots show below, the latter two methods lead to the most conservative estimates. If a single fold has an inflated R^2^, the first method of calculating R^2^ may lead to inflated estimated of overall R^2^ between observed and predicted expression, with this inflation more likely at smaller sample sizes.

Lastly, our ROS/MAP sample is a subset of the full ROS/MAP sample that has both genotype and expression data, also contributing to differences in the number of genes predicted above this R^2^ threshold. We note here that, adjusting our local window to 1 Megabase, rather than the original 0.5 Megabase, led to more significant expression models in both TCGA-BRCA and ROS/MAP.

**7. Similar number of genes with training R2>0.01 were also identified for the transcriptomic profiles of breast tissue type. This is also much less than what was published for studying breast cancer using TCGA data (**[**https://www.nature.com/articles/s41588-018-0132-x**](https://www.nature.com/articles/s41588-018-0132-x)**). The number of significant TWAS genes were also much less than published for using the same BRCA GWAS data set.**

There are a couple of points here: the reviewer is correct in pointing out that the number of TWAS genes we identify is much lower than in the Wu *et al* paper. However, there is an important distinction here: Wu *et al* conducted TWAS for **breast cancer susceptibility**, while we consider **breast cancer survival**, a specific research focus for our group in breast cancer outcomes (see Bhattacharya *et al* 2020, *Genome Biology* for a thorough motivation of this research focus). Another important caveat here is that Wu *et al* uses a small sample of healthy mammary tissue specimens from GTEx (N = 67), not TCGA tumor tissue, as we do in our analysis. Wu *et al* used TCGA tumor-adjacent normal samples for external validation of these models. Furthermore, there is no mention of a preliminary step for expression heritability estimation and an adjustment for sample size in the calculation of predictive R^2^. These may be disease-specific details for a methods paper, but we believe that these are imperative to consider.

First, tumor tissue may not be the best reference tissue to detect gene-trait associations for breast cancer susceptibility. Breast tumor expression is assayed after the initial diagnosis of breast cancer and is thus influenced by development and progression of the disease. This temporality may induce some bias in detecting associations; see Wainberg *et al* 2019, *Nature Genetics* for a discussion of choosing a proper tissue reference. Second, breast cancer risk and outcomes (e.g., survival or recurrence) are not necessarily associated with the same set of genes/genetic variants. There is a series of papers by Slattery *et al* from the Breast Cancer Health Disparities Study for differences in associations across risk and survival for genetic variants. We would not expect to see similar genetically regulated expression associations across breast cancer risk and survival; for example, see the differences between TWAS gene-trait associations for risk (Wu *et al* 2018, *Nature Genetics*) and survival (Bhattacharya *et al* 2020, *Genome Biology*). Third, studies of survival generally require larger numbers of samples (and thus events) compared to studies of risk to achieve an effect size of comparable magnitude. For these reasons, we believe that the reviewer’s comparison between our study of breast cancer survival and Wu *et al*’s study of breast cancer risk is not directly applicable.

**8. A discussion about which TWAS method (MeTWAS or DePMA) would be preferred by what type of genetic architecture, or multi-omics network for the test gene will be helpful.**

We thank the reviewer for highlighting the need for further discussion around the role of genetic architecture in method performance. We added an eQTL analysis of three genes in TCGA-BRCA at the end of the Results section: *LQK1* (high CV R^2^ with both MeTWAS and DePMA), *C6orf94* (high R^2^ only with MeTWAS), and *TSC22D4* (high R^2^ only with DePMA). Here, we saw that *LQK1* bears a strong local-eQTL signal that is leveraged by both models. In *C6orf94*, we find that eQTL signal is distributed evenly across the genome and the superior MeTWAS model leverages clusters of distal-eQTLs that are local to gene-associated mediators. In contrast, the distal-eQTL signal for *TSC22D4* is concentrated in a single peak with linkage disequilibrium support. This eQTL analysis provides some insight into genetic architectures that favor one MOSTWAS method over the other.

We also provide some intuition in the Discussion about scenarios where MeTWAS or DePMA is preferable with some ideas about tissue-specificity:

“...MeTWAS, a two-step regression method that leverages SNPs local to gene-associated mediators that are heritable, is preferred for genes that are influenced by a complex network of molecular features across the genome. Perhaps, a tissue-wide approach that searches for distal eQTL signals conserved across the genome could be an extension for MeTWAS [67]. In contrast, DePMA, which prioritizes significant distal-eQTLs with large mediation effects on the gene, may perform better when distal eQTLs that have strong LD support and are local to regulatory machinery have indirect effects on transcription of a gene. These distal eQTLs with large mediated effects may be tissue-specific, as they tend to huddle around epigenomic artifacts [68–70].”

**9. Color legend need to be included for Figure 4A, 4B.**

Thank you for pointing this out. We have added legends for these subfigures.
