# Supplementary material for: MOSTWAS: Multi-Omic Strategies for Transcriptome-Wide Association Studies
Source: PLoS Genet. 2021 Mar 8;17(3):e1009398. doi: 10.1371/journal.pgen.1009398 (PMC7971899; doi:10.1371/journal.pgen.1009398)
Supplement: Attachment — Submitted filename: Response to Reviewers.docx [file pgen.1009398.s021.docx]

January 20, 2021

Dear Dr. Zhu and Dr. Balding,

Enclosed is a response to reviewers for our manuscript:

*"****MOSTWAS: Multi-Omic Strategies for Transcriptome-Wide Association Studies****"*

We were happy to see that the reviewers were, on the whole, satisfied with our revised manuscript. In this revision, we have addressed some minor outstanding comments with no new analyses. In summary, we have modified Figure 4 to remove some annotations, included some language to guide readers in the methods overview section of the Results, and some caveats in our comparison of BGW-TWAS and MOSTWAS.

We believe that this revision has greatly improved our manuscript and thank the editors and reviewers for their helpful feedback.

| Arjun Bhattacharya  Postdoctoral Fellow  Department of Pathology and Laboratory Medicine  University of California, Los Angeles | Michael I. Love  Assistant Professor  Department of Biostatistics  Department of Genetics  University of North Carolina at Chapel Hill |
| --- | --- |

**Below is a point-by-point response to Reviewer 1 for our manuscript:**

We thank the reviewer for their helpful and thoughtful comments.

**Below is a point-by-point response to Reviewer 2 for our manuscript:**

Reviewer comments are bolded, and responses from the authors are indented.

1. **For the R2 comparison between BGW-TWAS and MOSTWAS, the figure showed there are more number of genes have greater R2 by BGW-TWAS method. It is not fair to compare the proportion of genes that have greater R2 as the total number of gene models fitted by BGW-TWAS method is >twice of the ones by MOSTWAS.**

The reviewer makes a fair point here, and accordingly, we have removed these annotations from Figure 4. This figure compares the external R^2^ of gene models passing inclusion criteria for both MOSTWAS and BGW-TWAS. Of these genes, MOSTWAS models show larger out-sample R^2^ for a large majority of genes.

We believe we have been fair to BGW-TWAS’s merits, as we write several times in our manuscript that BGW-TWAS produces more gene models than MOSTWAS and more models with R^2^ > 0.01 in external data (see Lines 242-243, 343-345, and 500). However, the reviewer has emphasized that BGW-TWAS produces more models than MOSTWAS; this is also not a fair one-to-one comparison. There are *vast differences* in the inclusion criteria for these two methods that we discuss in two sections in the manuscript. In the Results section (Lines 239-242), we write:

“Here, we note that BGW-TWAS uses a different inclusion criteria that MOSTWAS, where a BGW-TWAS model is trained if at least one eQTL with non-zero effect and sufficiently large posterior causal probability is detected without heritability estimation or cross-validation performance [31].”

In the Discussion section (Lines 474-476), we write:

“MOSTWAS’s stricter inclusion criterion (thresholds for both expression heritability and cross-validation performance) may lead to fewer genes trained and ultimately tested…”

We also, as discussed below, mention that incorporating other model fitting methods, like TIGAR’s DPR, could improve performance of MOSTWAS models and increase the number of models that pass MOSTWAS’s inclusion criteria.

2. **Please note that the BGW-TWAS computation time include single-variant eQTL test for genome-wide genotypes.**

We have included this note in the Discussion paragraph (Lines 481-482):

“Note that BGW-TWAS computation time includes genome-wide single-variant eQTL tests.”

3. **I think MOSTWAS might have an advantage over BGW-TWAS by extensively testing the mediation effect for some genes, but might miss some mediating effects that are too weak to be detected. The number of gene models fitted by MOSTWAS now is approximately similar to the so called local method but smaller than the BGW-TWAS method, which is mainly due to the Elastic-Net regression model. This could be improved if the Bayesian DPR model as used by TIGAR is used to fit the gene expression prediction model.**

We have included this consideration in the Discussion paragraph that compares BGW-TWAS and MOSTWAS (Lines 508-510):

“Alternative methods of fitting the predictive model, like TIGAR’s Dirichlet Prior Regression, could be built into the MOSTWAS framework to boost predictive power [40].”

However, we also contend that the discrepancy in the number of “significant models” is mostly a function of the large differences in inclusion criteria. We direct the reviewer to the points we make in the Discussion section about the importance of these inclusion criteria (Lines 502-508):

“MOSTWAS’s stricter inclusion criterion (thresholds for both expression heritability and cross-validation performance) may lead to fewer genes trained and ultimately tested; for example, when we subjected BGW-TWAS models trained with TCGA-BRCA data to cross-validation, few met MOSTWAS’s inclusion criterion of cross-validation adjusted $R^{2}>0.01$. We believe that assessing expression heritability signal and cross-validating models prior to TWAS is necessary: as Ding *et al* points out in their systematic power analysis, expression heritability is a major factor in determining TWAS power [82].”

4. **Since MOSTWAS may not be able to screen all other genome-wide multi-omics factors for each test gene, it would help to discuss how to select a set of “mediating omics factors” to start. And make it clear that if users need to first conduct single variant eQTL analysis for genome-wide genotypes per gene to identify all potential distal eQTL for mediation test?**

This is a good point. In the overview of the model in the Results, we have included this note for clarification. We also include some discussion about adding *a priori* knowledge into the list of mediators used in MeTWAS. We add in the Results section (Lines 130-134):

“For both methods, individual-level genotype data and omic data for mediators and genes of interest are required. Further, MeTWAS requires a list of mediators associated with the gene of interest. This list can be generated from distal-QTL analysis between mediators and genes or through *a priori* knowledge about a particular tissue. DePMA requires distal-eQTL summary statistics and local-xQTL summary statistics between SNPs and mediators.”

5. **I feel like a simplified method section could go ahead of the Results section to help reader to understand the method and results better. And more details could go to supplement. The editors can see if this would make sense.**

We have added some more language into the section of the Results that gives an overview of MOSTWAS using the biological mechanism as a backdrop. We believe that these additions, with the inclusion of the algorithmic summaries in Figure 1A-B, provides an intuitive explanation of MOSTWAS. This format also stays in accord with traditional PLOS Genetics guidelines.
